# Synthesis and characterization of trivalent cations (Fe_2_O_3_, Al_2_O_3_, and B_2_O_3_) incorporated into Ca_2_SiO_4_ bioceramics: bioactivity, optical, and antibacterial properties

**DOI:** 10.1039/d6ra04417d

**Published:** 2026-07-29

**Authors:** H. Agourrame, F. Amor, E. Ait-Ouakrim, M. Dahhou, N. Khachani, A. Zarrouk

**Affiliations:** a Laboratory of Applied Chemistry of Materials, Center for Materials Science, Faculty of Science, Mohammed V University in Rabat Avenue Ibn Battouta BP 1014 Rabat Morocco hind.agourrame@gmail.com +212615050686; b Microbiology and Molecular Biology Team, Center of Plant and Microbial Biotechnology, Biodiversity, and Environment, Faculty of Sciences, Mohammed V University in Rabat Rabat Morocco; c Medical Bacteriology Laboratory, National Institute of Hygiene Rabat Morocco; d Laboratory of Materials, Nanotechnologies and Environment, Mohammed V University of Rabat Morocco; e Laboratory of Molecular Spectroscopy Modelling, Materials, Nanomaterials, Water and Environment, CERNE2D, Faculty of Sciences, Mohammed V University in Rabat Morocco azarrouk@gmail.com +00212665201397

## Abstract

Herein, divalent cations such as Ca^2+^, Sr^2+^, and Mg^2+^ are widely studied for their contribution to the bioactivity and performance enhancement of biomaterials intended for bone applications. The incorporation of functional ions into these bioceramics thus represents a promising and economically viable approach. In contrast, Fe, Al, and B cations, although recognized for their influence on structure, chemical stability, and certain physicochemical properties, remain relatively unexplored in relation to the bioactivity of bone-related biomaterials. The present study focuses on the development of three bioactive bioceramics, successfully synthesized by the conventional solid-state reaction method, followed by heat treatments between 100 °C and 1000 °C. These bioceramics are based on the dicalcium silicate phase (Ca_2_SiO_4_), into which bioactive trivalent cations, such as Boron oxide (B_2_O_3_), Alumina (Al_2_O_3_) and Hematite (Fe_2_O_3_) have been incorporated, named CSF, CSA, CSB respectively. Furthermore, the bioactivity of these bioceramics were evaluated by immersing them in simulated body fluid (SBF) and artificial saliva (AS) for 24 hours at 37 °C. The samples were then characterized by powder X-ray diffraction (XRD), scanning electron microscopy (SEM), energy-dispersive X-ray spectroscopy (EDS), Fourier transform infrared spectroscopy (FT-IR), and UV spectroscopy. These analyses were complemented by using transmission electron microscopy (TEM). X-ray diffraction (XRD) results confirmed that the Dicalcium silicate (Ca_2_SiO_4_) phase is predominant in the three bioceramics, with secondary bioactive phases such as Grossite (CaAl_4_O_7_), Tricalcium borate (Ca_3_B_2_O_6_) and Brownmillerite (Ca_2_Fe_2_O_5_), specific to each incorporated oxide. The optical band gap of the samples, determined by UV-visible spectrophotometry, ranged from 3.60, 3.21 and 2.99 eV for CSF, CSB, CSA respectively. Antibacterial activity, evaluated against Gram-negative (*Pseudomonas aeruginosa*) and Gram-positive (*Staphylococcus aureus* and *Enterococcus faecalis*) bacteria, revealed significant inhibition of *Staphylococcus aureus*. XRD analyses after only 24 hours immersion in simulated media, complemented by scanning electron microscopy (SEM) observations, showed that these bioceramics rapidly promote the formation of a bone-like hydroxyapatite (Ca_10_(PO_4_)_6_(OH)_2_) layer, demonstrating their potential as biomaterials for bone regeneration. The results obtained indicate that the incorporation of bioactive oxides into dicalcium silicate may significantly improve its properties, thus enhancing its potential for tissue regeneration applications.

## Abstract 2

Herein, divalent cations such as Ca^2+^, Sr^2+^, and Mg^2+^ are widely studied for their contribution to the bioactivity and performance enhancement of biomaterials intended for bone applications.

## Graphical abstract

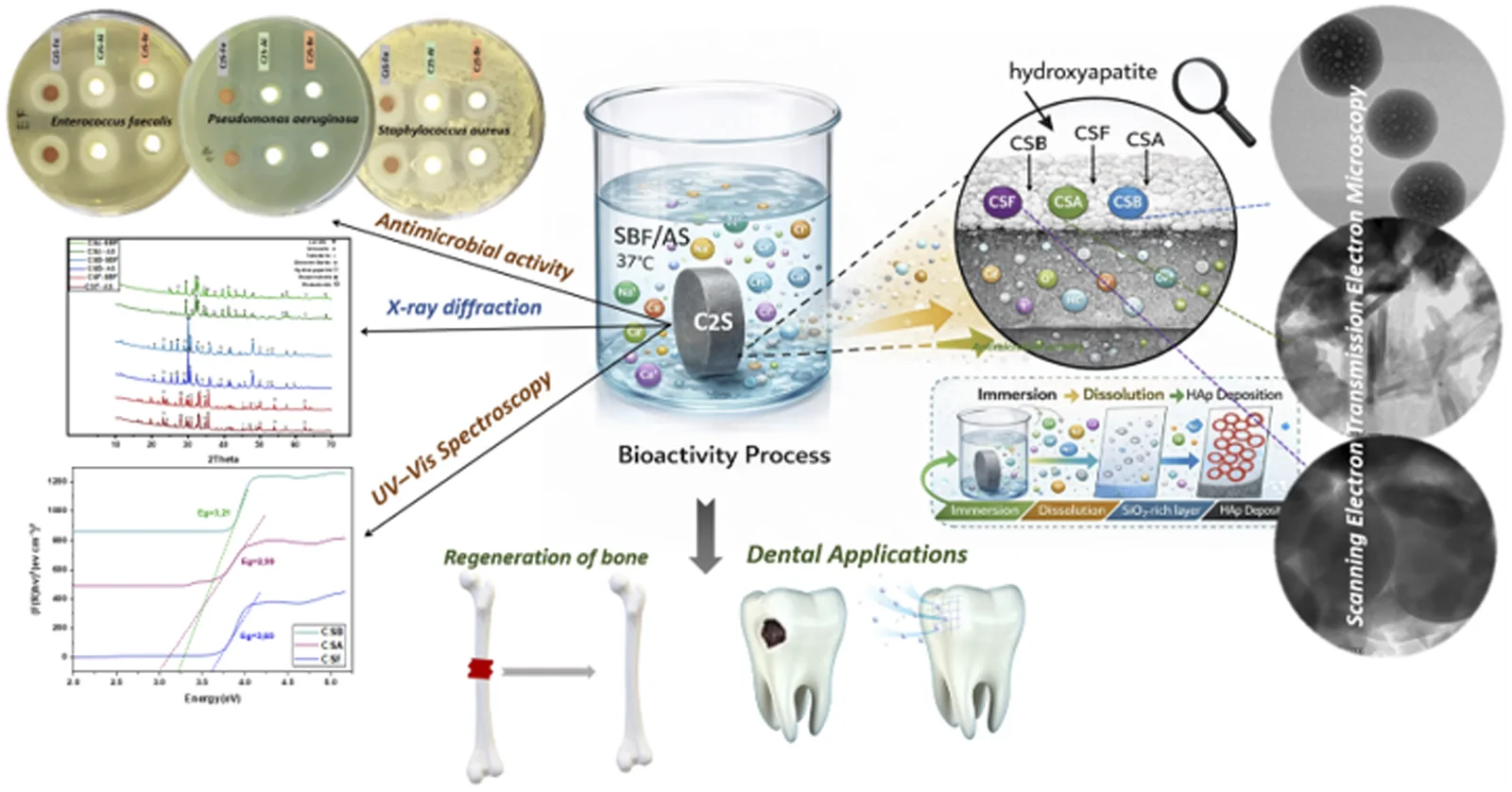

## 1. Introduction

Bioactive ceramics were recently discovered and have since given rise to new strategies as artificial materials for clinical bone repair and replacement.^1^ Ceramic materials used in restorative dentistry possess specific properties such as durability in the oral environment and similarity to the natural tooth structure.^2^ Their inertness to biological environments and high strength allow these materials to be used successfully for the fabrication of porous structures intended for implantation in humans.^3^ Bioactive ceramics bond and integrate spontaneously with living bone in the body, without the formation of fibrous tissue around them.^1^ These resorbable materials can be dissolved after the time required to fill the defect with new bone, without any toxic effects on human life. On the other hand, these bioabsorbable materials, which degrade and are absorbed in the human body, are useful as sutures for fixing bone fractures and as vectors for drug delivery.^3^ Hydroxyapatite (HA), with the chemical formula Ca_5_(PO_4_)_3_OH and a Ca/P molar ratio of 1.67, belongs to the calcium phosphate group and is considered a reference bone substitute. Highly biocompatible and non-toxic, it does not elicit a defensive reaction and interacts harmoniously with living tissues, constituting a major component of bone and dental tissue.^4,5^ One of the essential characteristics of a bioactive material is its ability to induce the formation of new HA layers *in vitro* when immersed in a simulated body fluid (SBF) with an ionic composition comparable to that of human blood plasma,^5^ or in dilute phosphate solutions.^6^ This ability promotes the physicochemical bond between the implant and living bone tissue, a crucial condition for osseointegration.^7^ Among these materials, bioactive ceramics exhibit for their high reactivity with physiological fluids and their ability to modify the surface, thus promoting the formation of a hydroxyapatite layer capable of ensuring strong adhesion to bone.^8,6^ The bioactivity mechanism comprises five observable *in vitro* steps, the first of which relies on ion exchange between the material and H_3_O^+^ ions from body fluid. This process induces the formation of a silica gel layer rich in silanol groups and a rise in pH, dependent on the material composition.^8^ The formation of this layer is highly dependent on the chemical composition of the material, which controls the release of soluble ions (Si, Ca, P, Na) that influence bioactivity mechanisms as well as intra- and extracellular cellular responses. Furthermore, doping with elements such as Cu, Zn, Ba, La, Y, Fe, Cr, or Sr allows for the adjustment and improvement of the material's bioactive properties.^8^ Numerous studies have recently focused on dicalcium silicate as a ceramic, distinguished by its high bioactivity, biocompatibility, and good mechanical properties, making it a promising material for bone implant applications.^9^ Dicalcium silicate (C_2_S) exists in several polymorphic forms, including α, α′, β, and γ. Of these, the β form is the most common in ordinary Portland cement, where it is stabilized by the presence of specific ions.^10^ Ca_2_SiO_4_ can be synthesized by various methods, including the sol–gel process,^11^ the Pechini method,^12^ combustion,^13^ hydrothermal synthesis,^13^ and spark plasma sintering.^14^ The stability of the β–C_2_S phase is highly dependent on the nature of the chemical stabilizing ions, such as B_2_O_3_, Al_2_O_3_, Na_2_O, K_2_O, BaO, MnO_2_, Cr_2_O_3_, or combinations thereof. The proportion of β–C_2_S formed varies according to the type of dopant ion and the stoichiometry of the sample composition.^15^ A previous study has reported that the Aluminum, in particular, incorporates itself into the dicalcium silicate structure by partially replacing the silicate units and creating oxygen vacancies, thus contributing to the stabilization of the β phase.^3^ A previous study reported that the incorporation of strontium into dicalcium silicate bioceramics enhances osteogenesis and suppresses bone resorption, thereby improving their potential for bone repair applications.^16^ Moreover, Dicalcium silicate (C_2_S) has the ability to incorporate chromium and vanadium, whose electrovalence states affect the stability and reactivity of its polymorphic phases under different redox conditions.^17^ In addition, boron doping and rapid cooling have been shown to stabilize the reactive α′H-C_2_S phase, enhance hydration performance, and markedly improve the mechanical strength of dicalcium silicate.^18^ Previous studies have shown that Fe doping modifies the reactivity of γ-dicalcium silicate by altering its local atomic structure and chemical bonding, with its overall effect depending on the reacting environment.^19^ Similarly, Ba^2+^ doping has been shown to activate β-dicalcium silicate by altering its crystal and electronic structures, resulting in improved hydration reactivity and early mechanical performance.^20^ The addition level of 8 wt% Fe_2_O_3_, Al_2_O_3_, and B_2_O was determined based on previous studies showing that moderate oxide incorporation can effectively modify the physicochemical and biological properties of calcium silicate bioceramics while maintaining β-Ca_2_SiO_4_ as the predominant phase. Previous studies have further revealed that the solid solubility of boron in β-C_2_S (2CaO·SiO_2_) is limited to approximately 0.309 wt%, whereas the solubility of Fe, expressed as FeO, in 2CaO·SiO_2_ is less than 2.8 mol%. Above these solubility limits, the excess oxides are expected to react during sintering to form secondary crystalline phases rather than being completely incorporated into the β-Ca_2_SiO_4_ structure.^21,22^ Although earlier studies reported the fight against antibiotic-resistant bacterial infections represents a major health challenge. In this context, inorganic nanomaterials, particularly metallic nanoparticles and metal oxides, are being studied as promising new antibacterial agents.^23^ Currently, some researches focused on role of iron oxide nanoparticles in the biomedical system. Many nanoparticles address technological advancements in the field of biological applications.^24^ Iron oxide (Fe_2_O_3_) nanoparticles are attracting increasing interest in the biomedical field due to their chemical stability, biocompatibility, and nanoscale size, which make them suitable for applications such as targeted therapy, tissue regeneration, hyperthermia, and anticancer, antiviral, and antibacterial approaches.^25^ These nanoparticles, particularly in their γ-Fe_2_O_3_ (maghemite) and Fe_3_O_4_ (magnetite) forms, are especially studied for their unique properties, such as paramagnetism, low toxicity, and high specific surface area, which are essential for tissue engineering.^26^ They also facilitate the delivery of therapeutic agents by increasing their permeability, stability, and circulation time,^27,28^ while allowing controlled drug release, thus reducing the risk of overdose and limiting side effects.^19,20^ Their ability to inhibit cell proliferation has been studied, in particular, in the treatment of breast cancer.^25^ Furthermore, the characteristics of these nanoparticles, such as their morphology, crystalline structure, and synthesis method, directly influence their performance.^29^ In tissue engineering, they can be integrated into scaffolds to support cell regeneration and locally release bioactive molecules that promote cell differentiation.^22,23^ Finally, Fe_2_O_3_-based nanocomposites also find applications in the fabrication of functionalized paramagnetic stents, notably for the treatment of myocardial infarctions and other cardiovascular diseases.^32^ Several studies have reported that the Hematite nanoparticles (α-Fe_2_O_3_) have demonstrated significant antimicrobial activity against various bacterial pathogens, generating increasing interest in their use in the development of environmentally friendly bionanomaterials for biomedical applications.^25,26^ Fe_2_O_3_ nanomaterials exhibit intrinsic antibacterial activity that improves with concentration against both Gram-positive and Gram-negative bacteria.^35^ Their activity is increased through synergistic and ROS-mediated mechanisms.^36,37,38,39^ Overall, they are potential antibacterial materials for biomedical applications.^40^ Among the metal oxides explored for their antibacterial properties, including ZnO, TiO_2_, CuO, and Fe_2_O_3_, the latter is characterized by moderate band gap (∼2.2 eV), its ability to absorb visible light (∼564 nm), its unique magnetic characteristics, and its good biocompatibility, making it a promising candidate for antimicrobial warfare.^41^ Whereas, boron, a non-metallic element belonging to group III of the periodic table, is abundant worldwide, with approximately 73.4% of identified reserves located in Turkey.^42^ It is currently considered a promising doping agent in bone tissue engineering, as it contributes to mineralization, bone growth, and the proper functioning of the central nervous system.^43^ In addition to its essential nutritional role, boron is actively being studied, along with magnesium, silver, and copper, to enhance the bioactivity, antimicrobial properties, and cell proliferation capacity of biomaterials.^44^ Its importance in bone development has also been confirmed by its direct effects on mineralization.^45^ Moreover, according to the literature, boron is known for its antibacterial and antifungal properties against a wide range of pathogenic microorganisms.^33,34^ However, its exact mechanism of action and its efficacy against certain specific species remain insufficiently elucidated.^46^ Studies have nevertheless shown significant antimicrobial activity of boron against Gram-positive bacteria such as *Staphylococcus aureus* and *Enterococcus faecalis*, as well as Gram-negative bacteria such as *Escherichia coli*.^47^ A previous investigation demonstrated that the calcium triborate phase (Ca_3_B_2_O_6_) also reveal antibacterial properties, effective even against antibiotic-resistant bacteria.^48^ Furthermore, alumina (Al_2_O_3_)-based materials are bio-inert ceramics widely used in the manufacture of joint and bone implants due to their excellent wear resistance and good mechanical properties.^49^ However, current knowledge of Al_2_O_3_-based composites remains limited, justifying the need for further research to overcome certain challenges and expand their biomedical applications.^50^ Progressive incorporation of Al_2_O_3_ has been shown to improve the bioactivity of materials, notably by promoting the accelerated formation of a hydroxycarbonate apatite layer on their surface when immersed in a simulated body fluid, an effect attributed to increased mechanical strength.^1^ In particular, controlled addition of Al_2_O_3_ has improved bone defect repair and controlled degradation rates, thus contributing to the long-term stability of implants.^51^ The improvement of the mechanical, thermal and chemical properties of alumina-doped bioglasses also enhances their suitability for use in demanding clinical applications.^1^ In addition, faced with the rise of antibiotic resistance, nanoparticles, particularly aluminum oxide (Al_2_O_3_) nanoparticles, is characterized by for their strong antimicrobial potential, determined by their nanometric size and large surface area.^52^ They have demonstrated effective antibacterial activity against Gram-positive and Gram-negative bacteria, including *Pseudomonas aeruginosa*, *Klebsiella pneumoniae*, and *Streptococcus aureus*, due to the formation of inhibition zones indicating their targeted action on bacterial membranes.^53^ Despite the widespread availability of aluminum and the numerous industrial uses of Al_2_O_3_ nanoparticles, their use in biomedicine, particularly for their antibacterial effects, remains underdeveloped.^23^ Moreover, an earlier study revealed that the calcium dialuminate (CaAl_4_O_7_), or grossite, is a mineral. Its monoclinic (*C*2/*c*) structure provides excellent thermal and chemical stability, while its tri-clusters enhance its crystallographic interest.^40,41^ It exhibits high transparency from the UV to the near-infrared and remarkable luminescence properties, making it promising for optical and functional applications.^56^ Its low cost and availability favor its use in optics, catalysis, and sensors.^54^ The incorporation of 8 wt% Fe_2_O_3_, Al_2_O_3_, or B_2_O_3_ was selected to enable a direct comparison of the effects of these bioactive oxides on the structural, bioactive, antibacterial, and optical properties of β-Ca_2_SiO_4_ bioceramics. This concentration was chosen based on previous reports demonstrating that moderate oxide incorporation can have a significant impact on calcium silicate ceramics while maintaining β-Ca_2_SiO_4_ as the predominant phase. The objective of this study was to performed a comparative study of the bioactivity of dicalcium silicate-based bioceramics incorporated with low percentages of bioactive oxides such as Fe_2_O_3_, Al_2_O_3_, and B_2_O_3_, by both *in vitro* immersion in artificial saliva and body fluid, to compare the development the formation of the hydroxyapatite layer on their surface. This study also examined their optical properties, analyzed by UV-Visible, Fourier transform infrared spectroscopy (FTIR), scanning electron microscopy (SEM), and Transmission Electron Microscopy (TEM). Furthermore, we demonstrated the significant influence of particle size on the bioactivity and chemical reactivity of these bioceramics, *via* the intensity of the functional groups.^30,31^

## 2. Experimental

### 2.1 Starting materials

The bioceramics Grossite –Dicalcium silicate (CaAl_4_O_7_–C_2_S), Calcium triborate–Dicalcium silicate (Ca_3_B_2_O_6_–C_2_S), and Brownmillerite–Dicalcium silicate (Ca_2_Fe_2_O_5_–C_2_S) were prepared *via* the solid-state reaction method. The synthesis was carried out using high-purity commercial powders of calcium carbonate (CaCO_3_, 99.99%) and silicon dioxide (SiO_2_, 99.99%). Low proportions (8%) of aluminum oxide (Al_2_O_3_), boron oxide (B_2_O_3_), or iron oxide (Fe_2_O_3_) were incorporated into the starting CaCO_3_–SiO_2_ mixture, while maintaining a Ca/Si molar ratio of 2. The finely homogenized mixtures were then followed by progressive heat treatment at 100 °C, 200 °C, 500 °C, 800 °C, and 1000 °C, with holding times of 1 h, 1 h, 4 h, 4 h, and 2 h, respectively, followed by rapid air cooling. To improve the reactivity of the precursors during heat treatment, intermediate grinding process were carried out in the presence of ethanol.

X-ray powder diffraction (XRD) of these samples was performed by a Bruker D2 X-ray diffractometer using Cu Kα radiation. The microstructure and elemental mapping of as-synthesized samples were recorded using scanning electron microscope (SEM, Jeol, 20KV) and Energy-dispersive X-ray spectroscopy (EDX) respectively. The High-resolution transmission electron microscopy (HRTEM) images were recorded using a field emission transmission electron microscope (JEOL). The UV–visible absorption spectra of the samples were recorded on a UV–visible spectroscope (JascoV-730).

### 2.2 *In vitro* bioactivity in artificial saliva and in simulated body fluid (SBF)

The bioactivity of a material corresponds to its ability to bind to bone *in vivo*, which can be assessed *in vitro* by the formation of a layer of hydroxyapatite phase in SBF medium, indicative of a future bone bond. The *in vitro* bioactivity of the prepared bioceramics (CSF, CSA and CSB) were evaluated by their ability to forming hydroxyapatite (HA) formation after immersion in both simulated physiological media: an artificial saliva (AS) solution and a simulated body fluid (SBF). The chemical composition of these both media is presented in Table 1. The artificial saliva used for the immersion tests corresponds to the formulations described by Carter-Brugirard and to the SAGF medium.^37^ The solutions were prepared by dissolving analytical grade reagents in ultrapure water, followed by pH adjustment to 6.8 using a 1 M hydrochloric acid solution. An alternative formulation of artificial saliva was also prepared by dissolving, per liter of distilled water. The pH of this solution was adjusted to 6.75 using orthophosphoric acid (H_3_PO_4_), a value representative of natural saliva. All the prepared solutions were place in polyethylene bottles.^44,45^ The bioceramic samples (0.5 g) were immersed in 15 mL of artificial saliva, placed in polyethylene vials, and then incubated at 37 °C for up to 24 h without agitation. At predetermined immersion times, the samples were retrieved, rinsed if necessary, and then dried at 60 °C for at least 24 h before being subjected to the various characterization analyses.

**Table 1:** Composition of the both artificial saliva and Simulated body fluid (SBF) solutions used in the study

Substances	Artificial saliva (g L^−1^)	Simulated body fluid (g L^−1^)
NaCl	0.125	8.03
KCl	0.963	0.22
CaCl_2_/CaCl_2_·2H_2_O	0.227 (CaCl_2_·2H_2_O)	0.29 (CaCl_2_)
MgCl_2_·6H_2_O	—	0.31
K_2_HPO_4_·3H_2_O	—	0.23
KH_2_PO_4_	0.654	—
NaHCO_3_	0.630	0.35
Na_2_SO_4_/Na_2_SO_4_·10H_2_O	0.763 (Na_2_SO_4_·10H_2_O)	0.07 (Na_2_SO_4_)
Urea	0.200	—
NH_4_Cl	0.178	—
KSCN	0.189	—

The simulated body fluid (SBF) solution was prepared and stored according to the protocol described in the literature. Bioactivity assays were performed by immersing 0.5 g of the prepared bioceramics in 15 mL of SBF, then incubating the system for 24 h in a water bath maintained at 37 ± 1.5 °C.^46,47,48^ The SBF preparation consisted of dissolving the various reagents in ultrapure water, strictly adhering to the dissolution order indicated in Table 1, with each salt being added only after the previous one had completely dissolved. Ionic stability and pH of the medium were maintained using buffering agents, namely TRIS.^63^ The final pH of the solution was adjusted to 7.40 at 36.5 °C by titration with 1.0 M HCl or 1.0 M NaOH solutions.^63^ The evolution of pH in SBF and AS are shown in Table 3. All bioceramics exhibited a gradual increase and stabilization in the range of approximately 6.2–8 after 24 h, attributed to early release ion exchange processes.^64^ The hydroxyapatite phase further evolution was analyzed (XRD, SEM and EDX) after 24 houres of soaking (Table 2).

**Table 2:** Abbreviation of the samples after 24 hours of immersion in artificial saliva (AS) and simulated body fluid (SBF)

	Bioceramics	CSF	CSA	CSB
Notation	After immersion the powders (CSF- CSA and CSB) in SA for 24 hours	CSF-SA	CSA-SA	CSB-SA
	After immersion the powders (CSF- CSA and CSB) in SBF for 24 hours	CSF-SBF	CSA-SBF	CSB-SBF

**Table 3:** pH of Simulated body fluid and artificial saliva after 24 h of immersion

Bioceramics	Simulated body fluid (initial pH = 7.40)	Artificial saliva (initial pH = 6.80)
CSA	7.88 ± 0.04	7.55 ± 0.03
CSF	7.91 ± 0.03	7.58 ± 0.04
CSB	7.82 ± 0.05	7.47 ± 0.05

### 2.3 Antimicrobial activity evaluation

The antibacterial activity of bioceramics CSF, CSB, and CSA incorporated with oxides, were evaluated by the Mueller-Hinton agar diffusion method. Bacterial suspensions of *Staphylococcus aureus*, *Enterococcus faecalis*, and *Pseudomonas aeruginosa* were prepared from recent cultures and adjusted to a turbidity of 0.5 McFarland, corresponding to approximately 1.5 × 10^8^ CFU mL^−1^. The surface of Petri dishes containing Mueller-Hinton agar was uniformly inoculated with each bacterial strain using a sterile swab. After the medium solidified, circular wells 6 to 8 mm in diameter were aseptically made in the agar using a sterile tip. The antimicrobial agent bioceramics CSF, CSB, and CSA were first dispersed in 1 mL of sterile DMSO to obtain a final concentration of 0.5 g mL^−1^. An appropriate volume of each suspension, corresponding to 0.5 g of material, was added to the prepared wells. The Petri culture plates were then incubated at 37 °C for 24 hours, allowing the antimicrobial agents to diffuse into the culture medium. After incubation, the antibacterial activity was assessed by measuring the diameter of the zones of inhibition (inhibition zone size was measured in millimeters) around each well. Each assay was performed in triplicate to ensure reproducibility of the results. Penicillin discs (30 µg, Sigma) were used as a positive control, while sterile DMSO served as a negative control. All experimental materials were sterilized by autoclaving at 120 °C for 20 minutes before use.

### 2.4 UV–vis spectroscopy

To determine the optical band gap of the studied bioceramics, the diffuse reflectance spectra were converted into pseudo-absorption functions *F*(*R*) using the Kubelka–Munk model, according to the expression *F*(*R*) = (1 – *R*)^2^/2*R*,^65^ where *R* denotes the absolute reflectance. Tauc diagrams were then established by plotting [*F*(*R*) *hν*]^*n*^ as a function of the photon energy *hν* (eV). The optical band gap *E*_g_ was estimated by linear extrapolation of the quasi-linear portion of the curves to the *x*-axis, according to the relation (*F*(*R*) *hυ*)^1/*n*^ = *A* (*hv* – *E*_g_),^66^ where *A* is a constant and *n* depends on the type of electronic transition. The values *n* = 1/2 and *n* = 2 correspond respectively to the direct and indirect transitions allowed, generally observed in bioceramics based on silicates and calcium phosphate.^67^

## 3. Results and discussion

### 3.1 X-ray diffraction

**Fig. 1:**
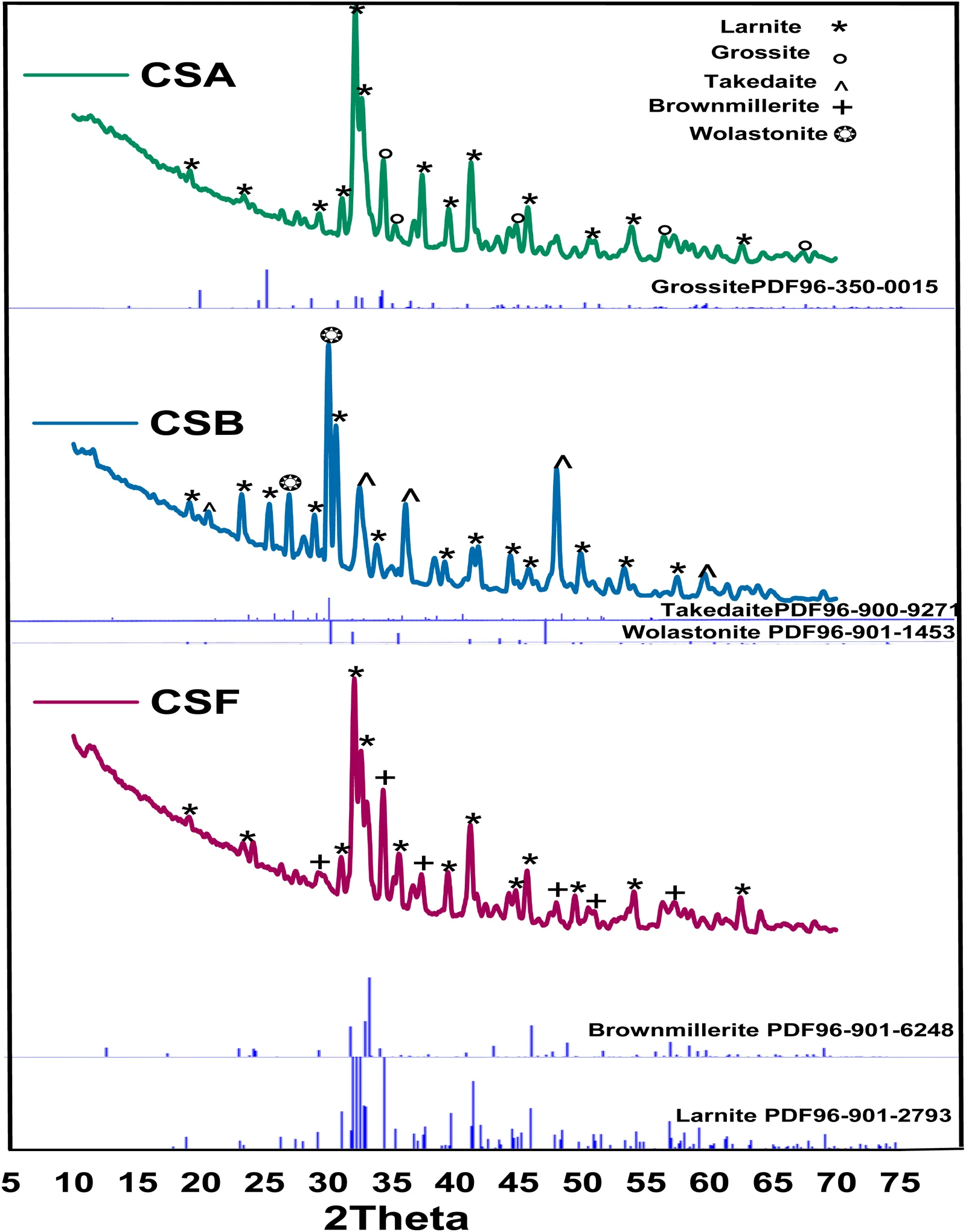
XRD diffractograms of CSF, CSB and CSA bioceramics.

**Fig. 2:**
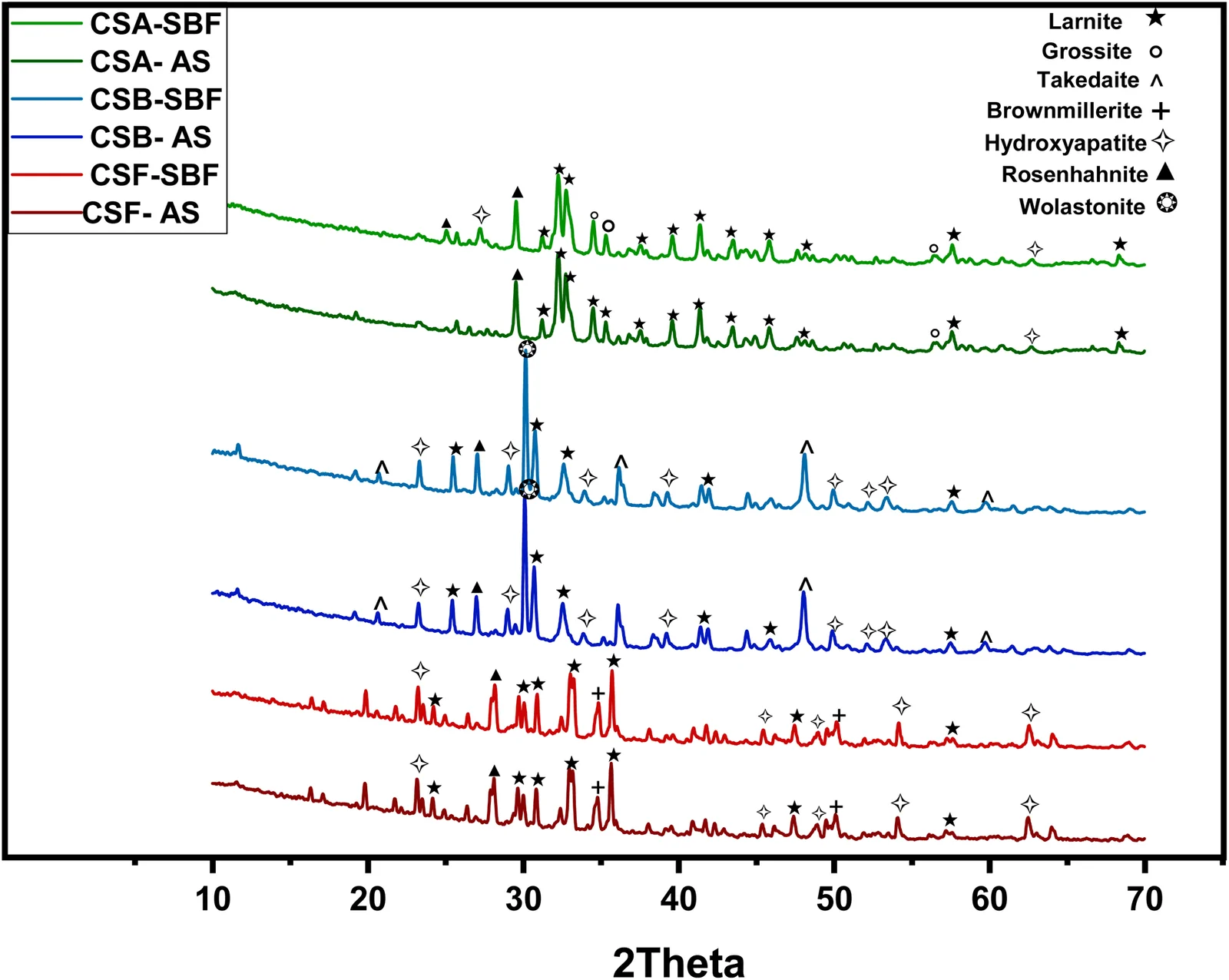
XRD diffractograms of bioceramics CSF, CSB and CSA after immersion in simulated body fluid (SBF) and in artificial saliva (AS).

Fig. 1 illustrates the XRD profiles of the CSF, CSB and CSA bioceramics following thermal treatment at 1000 °C. The XRD patterns reveal the formation of the Dicalcium silicate phase (β-Ca_2_SiO_4_, JCPDS datasheet no. 96-901-2793) in all synthesized samples. The identification of this phase was initially accomplished by referring to the standard JCPDS files, which provide references to standard compounds. In addition, the XRD patterns show that as secondary phases, as indicated by their low-intensity diffraction peaks, while β-Ca_2_SiO_4_ remained the dominant phase. These phases are also completely in accordance with standard data received from the Joint Committee on Powder Diffraction Standards and attributed to Grossite (CaAl_4_O_7_, JCPDS datasheet no. 96-350-0015) in CSA sample,^68^ Tricalcium borate (Ca_3_B_2_O_6_, JCPDS datasheet no. 96-900-9271) and Wolastonite (CaSiO_3_, JCPDS datasheet no.96-901-1453) phases in CSB sample, and orthorhombic Brownmillerite (Ca_2_Fe_2_O_5_) in CSF sample, belonging to the *Pnma* space group (JCPDS datasheet no. 96-901-6248).^69^Fig. 2 shows the powder X-ray diffractograms of the bioceramics obtained after immersion for a 24 hours in SBF and AS media, the pattern of the bioceramics contained peaks corresponding to Hydroxyapatite phase ((Ca_5_(PO_4_)_3_(OH)), JCPDS datasheet no. 99-100-1253), confirming the formation of an apatite layer on the surface of the bioceramics in both media. Furthermore, the presence of a few overlapping peaks is attributed to calcium silicate hydrates (Rosenhahnite, Ca_3_Si_3_O_8_(OH)_2_). Hence, these results confirm the apatite-forming ability of the Dicalcium silicate-based bioceramics in both physiological and simulated oral environments, with the B_2_O_3_ incorporated composition exhibiting more pronounced apatite deposition than the Fe_2_O_3_- and Al_2_O_3_-incorporated compositions.

### 3.2 Spectroscopy FTIR

**Fig. 3:**
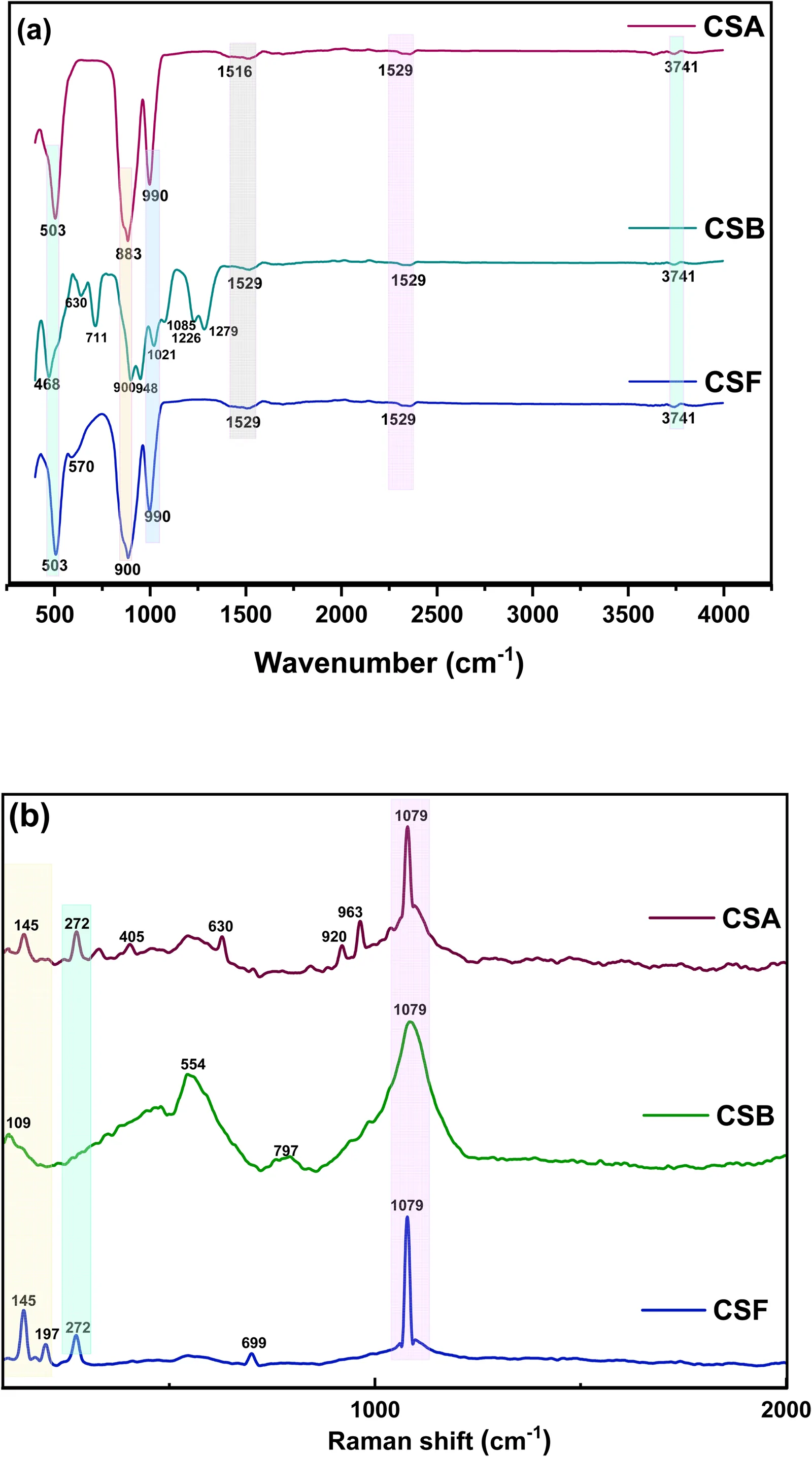
FT-IR spectra (a) and Raman Spectroscopy (b) of CFS, CSA, and CSB bioceramics.

The FTIR spectra were recorded to analyse the existence of vibrational modes, functional groups and bonds in the prepared bioceramics. The FTIR spectrum of CFS, CSA, and CSB samples are shown in Fig. 3a. The FTIR analysis shows that the three bioceramics have similar functional groups, the differences observed relating mainly to the intensity of the bands. The most intense bands located at 800 and 1000 cm^−1^ correspond to the symmetric and asymmetric vibrations of the Si-O bonds present in the dicalcium silicate structure.^55,56^ Besides, the bands located at the regions at 1150–1600 cm^−1^ and 800–1150 cm^−1^ were arisen due to the asymmetric nature of both the triangular BO_3_ and tetrahedral BO_4_ units, respectively.^60,62^ Thus, the absorption peaks between 700 cm^−1^ and 615 cm^−1^ are associated with the bending of the B–O and B–O–B unit with oxygen atoms in the BO_3_ and BO_4_ out of the plane blending modes.^57,58,59^ Additionally, the existence of smaller peaks Fig. 3a centered around 570 cm^−1^ shows the existence of FeO bond stretching in CSF sample.^75^ The decrease in the intensity of certain bands in CSB sample indicates that the addition of B_2_O_3_ in the dicalcium silicate decreased the intensity of Si–O vibrations, compared to their incorporated with Al_2_O_3_ and Fe_2_O_3_.^72^ Therefore, results from FTIR spectra confirm with those from the previous XRD analysis. The Raman spectrum of the bioceramics Fig. 3b reveals several characteristic vibrational bands associated with the crystalline phases. The band observed at 554 cm^−1^ is assigned to the symmetric Fe–O stretching vibration of FeO_4_ tetrahedra, confirming the presence of the Brownmillerite (Ca_2_Fe_2_O_5_) phase.^76^ The bands located at 424 cm^−1^ and in the low-frequency region (<300 cm^−1^) are attributed to Ca^2+^/Ca–O lattice vibrations.^77^ In addition, the band at 540 cm^−1^ corresponds to the symmetric Al–O stretching vibration, indicating the presence of the Grossite (CaAl_4_O_7_) phase.^78^ The Raman band observed at approximately 900 cm^−1^ is assigned to the asymmetric B–O stretching vibration of BO_3_ groups, characteristic of Tricalcium borate (Ca_3_B_2_O_6_).^79^

### 3.3 Scanning electron microscopy (SEM)

**Fig. 4:**
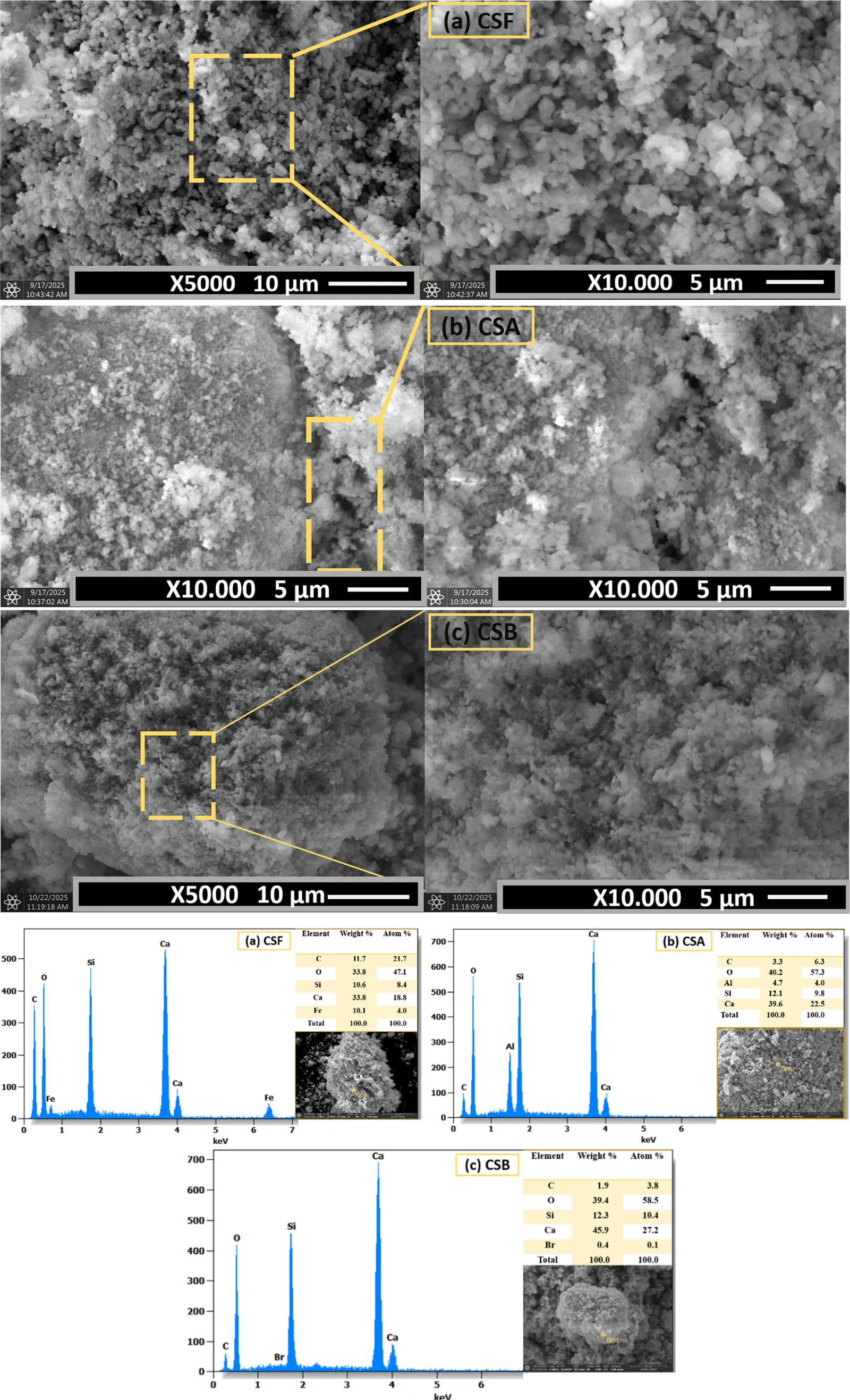
SEM (a–c) and EDS (a–c) of CFS, CSA, and CSB bioceramics ×1 µm and ×5 µm.

**Fig. 5:**
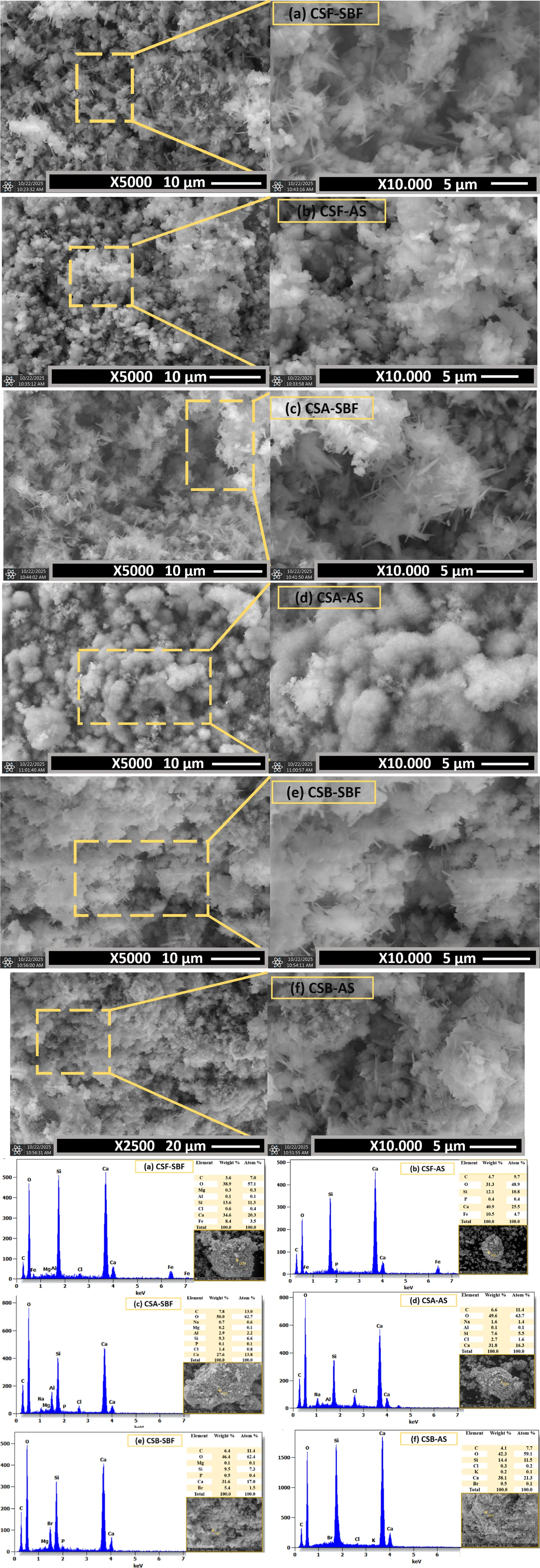
SEM (a–f) and EDS (a–f) samples after immersion in simulated body fluid (SBF) and in artificial saliva (SA) ×10 µm and ×5 µm.

The figures shows the representative SEM images corresponding to bioceramics at different magnifications. SEM was used to study the morphological changes in the bioceramics before and after incubation in artificial saliva (AS) and in simulated body fluid (SBF). Fig. 4 shows the SEM images and EDS spectra of the bioceramics surfaces before immersion in SBF and AS. The variety of morphologies and shapes were observed after immersion in simulated body fluid and artificial saliva, all samples exhibited a notable morphological change, characterized by the appearance of surface deposits and a granular layer, attributed to the formation of a hydroxyapatite phase. The SEM images (Fig. 4a) reported an inhomogeneous structure formed by agglomeration of irregular shaped grains.^75^ Thus confirming the presence of Brownmillerite (Ca_2_Fe_2_O_5_) phase,^75^ a combined rod and sphere shape morphologies were observed in the bioceramic indicates the presence of β-C_2_S phase. This was explained with the co-existence of two morphology on bioceramic. At a greater magnification (Fig. 4b), in the case of CSA, The observed grains result from the agglomeration of several particles, giving the particles an irregular morphology.^53,61^ With clearly defined grain boundaries confirm the presence of Grossite (CaAl_4_O_7_).^54^ In the case of CSB (Fig. 4c) sample the grains can be clearly observed on the surface of the bioceramic. It is obvious that the surface of the particles is very smooth and clean.^81^ On the other hand, exhibit irregular shapes. The some particle have particle size in the range of less than 20 nm. Thus, it can be said that although the particles are in nanometer range, but the shape and dimension of particle distribution are irregular shapes and aggregate each other reveal the presence of Tricalcium borate (Ca_3_B_2_O_6_).^72^ In Fig. 4, the atomic percentage depending on the chemical element for the bioceramics. These results were obtained using EDS technique. According to these results, the predominant elements in the bioceramics are Ca, Si, B, Al, Fe and O. Fig. 5 shows SEM micrographs for bioceramics, respectively submerged in SBF and SA during 24 hours by a cross-sectional observation and EDX analysis of their top-surface. For the bioactivity analysis, the bioceramics were synthesized and after that, they do not exhibit the same powders morphology. However, microscopic observation reveals that both samples CSA and CSF shows a significant change in their morphology after immersion in body fluid, evolving towards a fibrous structure, formed and covered most areas of the CSA – SBF surfaces.^83^ SEM analysis of the surfaces of the CSF- SA and, CSF- SBF (Fig. 5a and b) showed that the morphology delivered very good homogeneity of the nanocomposite, even when clusters of nanoparticles would usually be expected.^84^ Even after in immersion in SBF (CSF- SBF), newly formed particles was observed on the surface the fibril-like structures had smaller and more uniform diameter with a randomly oriented microstructure.^66,67^ As shown in (Fig. 5c and d), the prepared bioceramics CSA- SA showed that had an initial uniform layer of submicron crystals with the increase in acicular crystals grouped as hemispherical clusters.^87^ The apatite growth exhibiting spherical shapes, can be observed. These spheres are agglomerated in order to form an interconnected lattice like ordering, the apatite formation was observed 24 hours after the immersion in SBF.^5^ Moreover, The EDS spectrum of the sample bioceramic indicates that it is mainly composed of Ca, Si, Al, P and O. In the same fashion, CSB-SA and CSB-SBF (Fig. 5e and f) bioceramics shows surface changes of the surface, as an higher increase can clearly be seen on the surface of the CSB-SA in artificial saliva comparing with CSB-SBF in SBF. Indicating low effect of artificial saliva media.^88^ However, the absence of the phosphorus element in the EDS spectra of the CSB-SA, CSA-SA, CSF-FCS bioceramics samples is attributed to its low amount concentration in artificial saliva and simulated body fluid to be detected by EDS analysis.^80,85,86^ Overall, SEM observations confirm that the incorporation of Al_2_O_3_, B_2_O_3_ and Fe_2_O_3_ oxides into Ca_2_SiO_4_ phase has a strong influence on the surface microstructure of dicalcium silicate, with a more pronounced bioactive response after immersion in simulated body fluid compared to artificial saliva, indicating active interaction the bioceramics with the biological environment. Elsewhere, the SEM micrographs indicate the formation of layered deposits of needle-like and plate-like.^87^ However, his further confirms with the deposit of amorphous apatite layer on the surface of all bioceramics.^89–91^

### 3.4 Antimicrobial activity

**Fig. 6:**
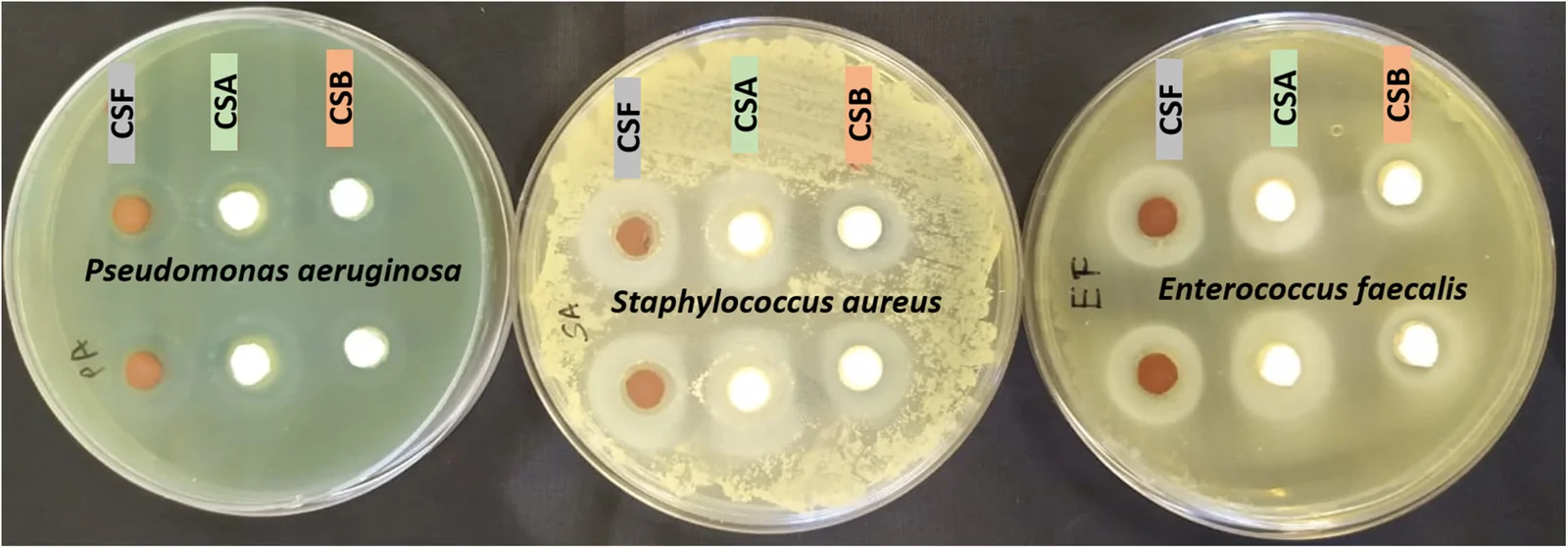
Antibacterial activity of CFS, CSA, and CSB extracts against *Staphylococcus aureus*, *Enterococcus faecalis* and *Pseudomonas aeruginosa*.

**Fig. 7:**
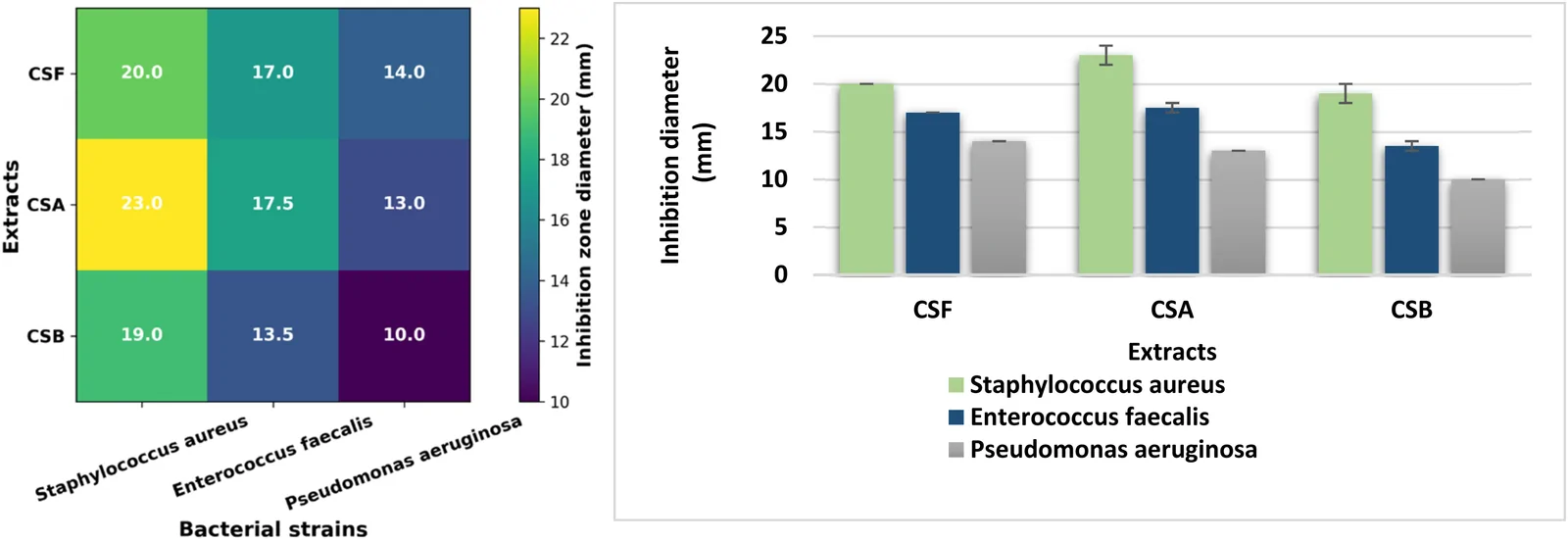
Heapt of inhibition zone diameters for CFS, CSA, and CSB extracts.

The results obtained reveal a higher antimicrobial activity of metal oxides towards Gram-positive bacteria (*Staphylococcus aureus* and *Enterococcus faecalis*) compared to Gram-negative bacteria (*Pseudomonas aeruginosa*). Bioceramic CSB containing B_2_O_3_ exhibited inhibition zones of 10 mm for *Pseudomonas aeruginosa*, 19 mm for *Staphylococcus aureus*, and 13 mm for *Enterococcus faecalis*. Although the inhibition of *Pseudomonas aeruginosa* was relatively moderate in this study. Moreover, many studies reveal that boron can effectively inhibit this bacterium at low concentrations by disrupting bacterial metabolism, membrane integrity, and intracellular ionic balance.^70,71^ High inhibition of *Staphylococcus aureus* and *Enterococcus faecalis* to boron-containing phases has also been reported, confirming the antibacterial potential of borated materials.^72,73^ Bioceramic CSA incorporated with Al_2_O_3_ demonstrated the most effective antibacterial activity, with inhibition zones of 13 mm for *Pseudomonas aeruginosa*, 23 mm for *Staphylococcus aureus*, and 17 mm for *Enterococcus faecalis*.^74^ These results confirm the antibacterial efficacy of Al_2_O_3_ nanoparticles reported in the literature, particularly their broad spectrum of activity against *Staphylococcus aureus*, *Pseudomonas aeruginosa*, *Escherichia coli*, and *Candida albicans*. According to previous studies represent that, the antibacterial activity of nano-Al_2_O_3_ is attributed to its strongly positive surface charge, which promotes electrostatic interactions with the negatively charged bacterial cell wall rich in lipopolysaccharides, leading to membrane adsorption, structural damage, and cell aggregation.^92^ Furthermore, the release of Al^3+^ ions contributes to the generation of reactive oxygen species (ROS),^93,94^ and increased membrane permeability, thereby disrupting bacterial enzymes and proteins.^23^ Regarding the CSF bioceramic, inhibition zones of 13 mm for *Pseudomonas aeruginosa,* 20 mm for *Staphylococcus aureus*, and 16 mm for *Enterococcus faecalis* were measured. These results are consistent with studies reporting significant antibacterial activity of iron oxide nanoparticles (Fe_2_O_3_), particularly against *Enterococcus faecalis*.^75,76^ The antibacterial effect of Fe_2_O_3_ is primarily based on the production of ROS and the release of soluble ions, leading to disruption of bacterial cell functions. It is also reported that excessively high concentrations can reduce antibacterial efficacy due to nanoparticle aggregation and electrostatic repulsion forces.^95^ Furthermore, several studies indicate that Fe_2_O_3_ predominantly exert a bacteriostatic rather than bactericidal effect, which may explain the moderate zones of inhibition observed in this study.^96^ Overall, antibacterial activity is dependent on the incorporated oxide and the type of bacteria, revealing specific interactions between the bioceramic surface and bacterial cell walls. The enhanced antibacterial activity of Fe-, Br-, and Al-doped dicalcium silicate bioceramics is attributed to the synergistic effects of the incorporated dopant ions. Fe- and Al-containing materials mainly act through reactive oxygen species (ROS) generation, electrostatic interactions with bacterial membranes, and the inactivation of essential cellular components, leading to damage the membrane disruption. Fe enhances ROS production *via* the Fe^3+^/Fe^2+^ redox cycle and improves charge separation,^97^ while Al contributes to ROS formation and membrane damage through electrostatic attraction, with its activity depending on the crystalline phase.^23^ Bromine-based compounds also exhibit broad-spectrum antimicrobial effects related to oxidative stress and membrane-associated mechanisms. In addition, Gram-negative bacteria are generally more susceptible than Gram-positive ones due to their thinner peptidoglycan layer, which facilitates nanoparticle penetration. Overall, the superior antibacterial performance results from combined oxidative stress, electrostatic interactions, and membrane disruption mechanisms.^98^ Although the pH of the immersion media increased after 24 h, it remained below 8.0. The antibacterial activity of dicalcium silicate based bioceramics is significantly affected by the alkalization of the immersion medium resulting from their dissolution. The release of Ca^2+^ ions, accompanied by the generation of OH^−^ ions, elevates the environmental pH and promotes that are unfavorable for bacterial growth. Reported studies have demonstrated that the antibacterial performance is directly related to the pH of the surrounding medium, confirming the important role of alkalinity in bacterial inhibition.^99^ It has also been reported that the antibacterial activity can be significantly reduced or completely suppressed after neutralization of the alkaline medium, confirming that pH plays a crucial role in the antibacterial mechanism.^100^ Furthermore, highly alkaline environments have been shown to effectively inhibit bacterial growth (Fig. 6 and 7).^101^

#### 3.4.1 Transmission electron microscopy

**Fig. 8:**
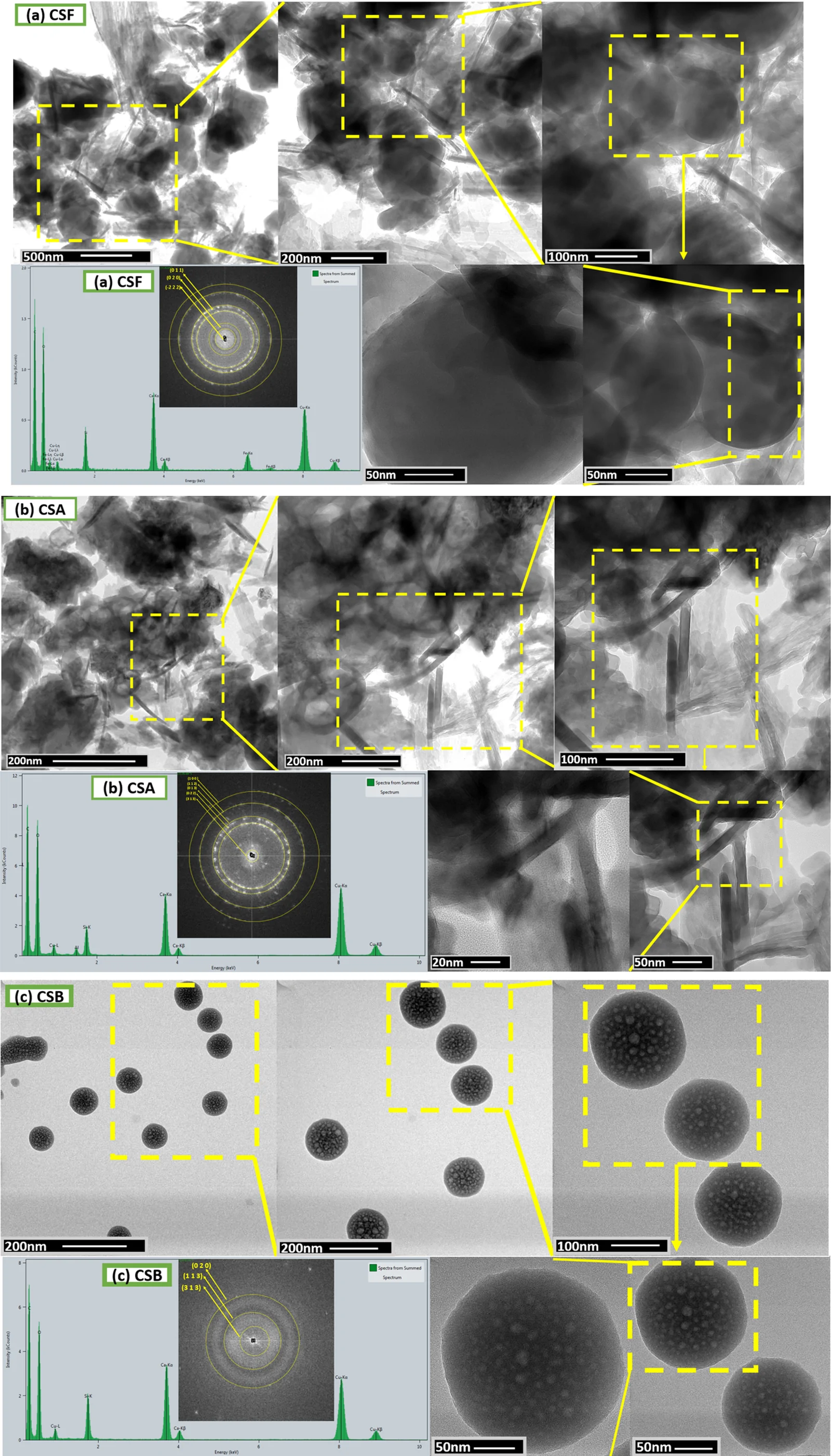
Transmission electron microscope (TEM) images of bioceramics (CFS, CSA, and CSB) nanoparticles (50 nm). (a–c) Selected diffraction pattern of the corresponding of nanoparticles. (a–c) High-resolution TEM image of the corresponding bioceramics nanoparticles (5 nm).

The morphology of bioceramics has been investigated by TEM observations. It is difficult to determine exactly the form of the particles by SEM. In order to obtain more reliable measurements of the morphology of the samples and to prove the co-existence of both phases within. The Transmission electron microscope (TEM) was carried out on the powder bioceramics to observe their particle average sizes and size distribution of the morphology. A notable common feature among the three samples is the observation of small spherical nanoparticles highly dispersed on the surface of the main particles indicates the presence of the dicalcium silicate phase (Fig. 8a) shows the TEM and HRTEM images of the prepared CSF bioceramic nanoparticles showed both distinct morphologies, which confirms the combination or mixture of both phases within the sample. The presence of the irregular shaped and larger sized agglomerated particles corresponds to the Brownmillerite (Ca_2_Fe_2_O_5_) phase.^102^ While, the β-C_2_S crystal observed in the bioceramic exhibits a predominantly spherical and rod-shaped morphology, which differs from the short acicular form generally reported in the literature.^103^ As discussed above, the XRD pattern of bioceramic indicates the presence of a Brownmillerite (Ca_2_Fe_2_O_5_) and β-C_2_S phases. From the bright field TEM image From the bright field shown in (Fig. 8c) it is clear that the coexistence of spherical particles with defined edges and larger grains observed on the surface of the CSB sample, it is clear that nearly monodisperse nanoparticles,^104^ are spherical in shape,^72,82,83^ reveals the formation of the phase Tricalcium borate (Ca_3_B_2_O_6_) phase, but they are connected to one another in many places to form agglomerated structure. Furthermore, the result indicates that TEM images show on whose surface on these spherical particles are dispersed small,^105,106^ also spherical, nanoparticles, indicating the presence of dicalcium silicate phase. In that respect, the sample incorporated by Al_2_O_3_ exhibits well-defined rods or platelets particles,^107^ ength interlocked together with the presence of particules exhibits the redrawn fibers form.^108^ However, associated with a few particles of platy and needle-like crystalline phases,^109^ reveal the presence of Grossite (CaAl_4_O_7_) phase. However, surface is completely covered with particles in the form of bud-like nanorods. The selected electron diffraction (SAED) patterns recorded for the three bioceramics (Fig. 8a–c) correspond to the diffraction of several particles and are characterized by the presence of dotted rings, typical of materials composed of small, well-crystallized grains. For each sample, indexing the diffraction points reveals the presence of the Grossite (CaAl_4_O_7_), Tricalcium borate (Ca_3_B_2_O_6_), and Brownmillerite (Ca_2_Fe_2_O_5_) phases, respectively, as shown in Fig. 8a–c. Furthermore, the measured interplanar spacings obtained from the SAED patterns are consistent with the corresponding crystallographic planes identified by XRD, providing additional confirmation of the assigned phases. These results are in good agreement with the results obtained by X-ray diffraction, thus confirming the crystalline nature of the synthesized bioceramics. Furthermore, the chemical composition of the three samples was verified by EDS analysis, confirming the presence of the expected constituent elements for each phase.

### 3.5 Bioactivity study

As reported by Kokubo and Takadama, the ability of a bioceramic to induce apatite formation after immersion in simulated body fluid (SBF) or artificial saliva (AS) is directly due to its capacity to establish a chemical bond with bone by the formation of a bioactive apatite layer.^110^ In the present study C_2_S-based bioactive oxides modified bioceramics, apatite formation is results from a sequence of interfacial physicochemical reactions occurring at the bioceramic solution interface. When immersion, partial dissolution of the calcium silicate, releasing Ca^2+^ ions into the medium while the Si–O–Si bonds is hydrolyzed. Concurrently, ion exchange between Ca^2+^ from the bioceramic and H^+^/H_3_O^+^ ions from the physiological solution promotes the formation of silanol (Si–OH) groups on the surface.^87^ Thereafter deprotonation of these silanol groups generates negatively charged silanolate (Si–O^−^) sites, which act as preferential nucleation centers for apatite formation. These negatively charged sites electrostatically attract Ca^2+^ ions from the solution, producing a calcium-rich surface layer that subsequently adsorbs phosphate (PO_4_^3−^) ions, leading to the precipitation of an amorphous calcium phosphate (ACP) layer.^111,112^ As a result, the SEM observations clearly support this mechanism, revealing the formation of an apatite layer on the surfaces of all investigated bioceramics after immersion in both SBF and artificial saliva. However, the CSB sample exhibited the most homogeneous, compact, and continuous apatite coating, followed by CSF and CSA bioceramic. These results are confirmed by the XRD and FTIR results, which revealed a more pronounced formation of apatite-related phases for CSB. The bioactivity of CSB can be attributed to the presence of minor calcium borate secondary phase, which enhance ionic dissolution and facilitate the formation of active nucleation sites, thereby accelerating apatite nucleation. Consequently, the bioactivity of the investigated bioceramics follows the order CSB > CSF > CSA in both SBF and artificial saliva.

### 3.6 UV–vis spectroscopy

**Fig. 9:**
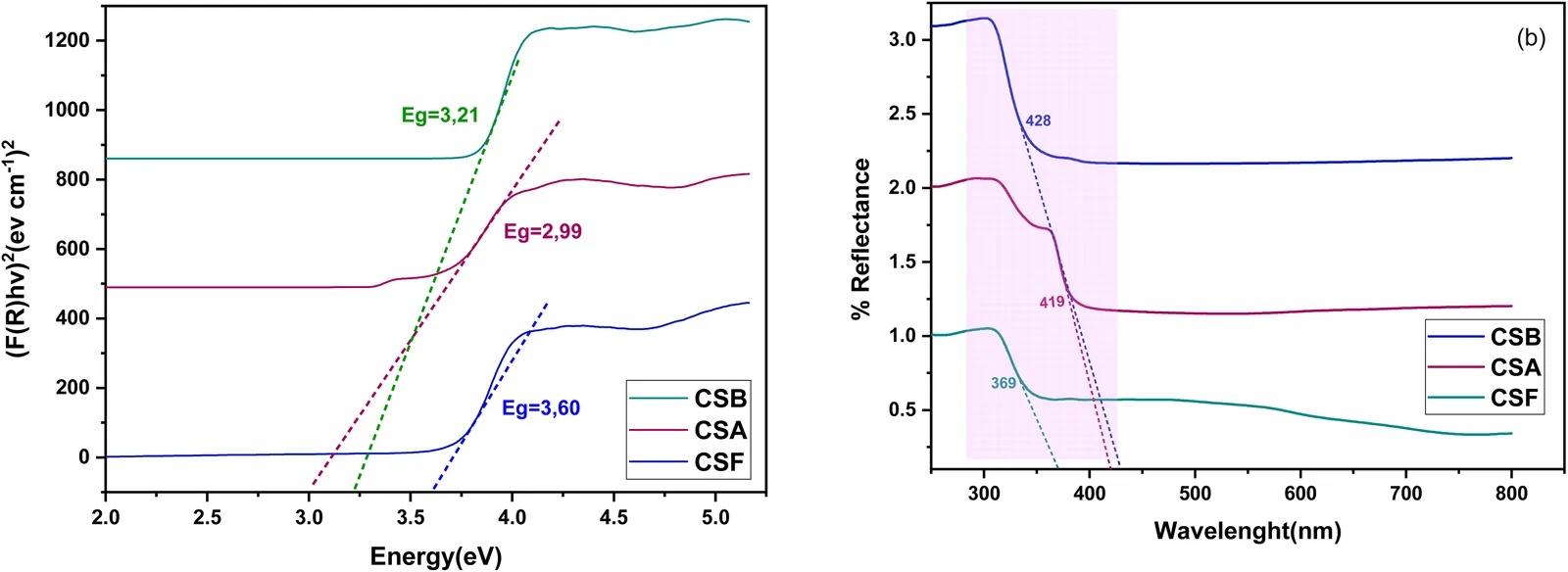
(a) The diffused reflectance spectra and (b) modified Kubelka–Munk plots of CFS, CSA, and CSB.

The UV-vis absorption of the prepared bioceramics of CSF, CSB and CSA are shown in (Fig. 9a and b). Dicalcium silicate (C_2_S) exhibits a high band gap energy (*E*_g_ = 5.127 eV).^113^ Noteworthy, the literature reveal the band gap energy values for the oxides studied, with optical gaps of approximately 6.14 eV for Al_2_O_3_,^114^ 6.20 eV for B_2_O_3_ (ref 115) and 5.0 eV for Fe_2_O_3_,^116^ respectively. The incorporation of aluminum oxide (Al_2_O_3_) induces a marked decrease in the band gap energy (Fig. 9a), which reaches a value of *E*_g_ = 3.22 eV, associated with an absorption edge observed at 419 nm (Fig. 9b). This significant reduction compared to pure C_2_S suggests the introduction of intermediate electronic levels within the band gap. Thus giving the bioceramic semiconductor behavior. On the other hand, the incorporation of B_2_O_3_ leads to an even more pronounced reduction in the band gap energy, with an *E*_g_ value of 2.94 eV (Fig. 9a) and an absorption maximum observed around 369 nm (Fig. 9b). In comparison, pure B_2_O_3_ exhibits a high band gap energy, around 6.20 eV according to the literature, confirming that its incorporation into the C_2_S matrix induces a significant decrease in the optical band gap. Whereas sample CSF show that the band gap energy increases slightly to *E*_g_ = 3.60 eV (Fig. 9a), with an absorption value located around 428 nm. The optical band gap of Fe_2_O_3_ is generally reported to be around 2.67 eV, indicating that its incorporation into the C_2_S structure induces a moderate band gap reduction. Overall, the UV–Vis results demonstrate that significant modifications in the optical properties depending on the oxide type. These findings emphasize that the CSB sample exhibit the lowest optical band gap energy among CSF et CSA. making it a more promising candidate for applications requiring efficient light absorption in the visible region.

## 4. Conclusion

In conclusion, dicalcium silicate (Ca_2_SiO_4_) incorporating the bioactive oxides boron oxide (B_2_O_3_), alumina (Al_2_O_3_), and hematite (Fe_2_O_3_) was successfully synthesized *via* the solid-state reaction method, demonstrating that this method effectively enables the formation of the corresponding bioceramic phases (CSB, CSA, and CSF). Furthermore, X-ray diffraction (XRD) analysis allowed us to observe that the three bioceramics studied exhibit an assembly of bioactive phases coexisting with the dicalcium silicate phase. Furthermore, from the FTIR results, typical functional groups of bioceramics were identified. Finally, SEM results indicated that the morphology of the materials is strongly influenced by the oxides type. Moreover, the employed characterization techniques used, in particular X-ray diffraction (XRD), confirmed the presence of the hydroxyapatite phase, while SEM-EDS analyses made it possible to establish a comparison among bioactive materials with different surface morphologies, in order to verify, for each of them, the nucleation mechanisms and the evolution kinetics of hydroxyapatite during the first 24 hours of soaking. Hence, CSF and CSA bioceramics incorporated with Fe_2_O_3_ and Al_2_O_3_ oxides exhibit notable antibacterial activity against Gram-positive and Gram-negative bacteria, while CSB bioceramic containing B_2_O_3_ shows significantly lower efficacy compared to other bioceramics. Thereafter, UV-Visible analysis shows that the C_2_S phase are moderately affected by the nature of the incorporated oxide. Based on the results obtained in the current study, it is possible to conclude that the three bioceramics exhibited *in vitro* mineralization in simulated body fluid (SBF) and artificial saliva (AS) solutions, constitute an alternative bioceramics to be considered for bone tissue engineering in future. Although the selected oxide content (8 wt%) successfully improved the physicochemical and biological performance of the bioceramics, future studies are required to evaluate its long-term cytocompatibility and biological safety before biomedical applications.

## Conflicts of interest

There are no conflicts to declare.

## References

[cit1] El-Kheshen A. A., Khaliafa F. A., Saad E. A., Elwan R. L. (2008). Effect of Al_2_O_3_ addition on bioactivity, thermal and mechanical properties of some bioactive glasses. Ceram. Int..

[cit2] Singh A. K., Bose S., Bandyopadhyay S. (2016). Effect of Al_2_O_3_ on leucite based bioactive glass ceramic composite for dental veneering. Ceram. Int..

[cit3] Mesquita-Guimarães J. , Souza J. C. M., Hotza D., Henriques B. and Boccaccini A. R., Nanostructured biocompatible ceramics and glass-ceramics, in Nanostructured Biomaterials for Cranio-Maxillofacial and Oral Applications, Elsevier, 2018, pp. 97–118

[cit4] Ravarian R., Moztarzadeh F., Hashjin M. S., Rabiee S. M., Khoshakhlagh P., Tahriri M. (2010). Synthesis, characterization and bioactivity investigation of bioglass/hydroxyapatite composite. Ceram. Int..

[cit5] Sossa P. A. F., Giraldo B. S., Garcia B. C. G., Parra E. R., Arango P. J. A. (2018). Comparative study between natural and synthetic hydroxyapatite: structural, morphological and bioactivity properties. Materia.

[cit6] Xu J., Wang Y., Huang Y., Cheng H., Seo H. J. (2017). Surface reactivity and hydroxyapatite formation on Ca_5_MgSi_3_O_12_ ceramics in simulated body fluid. Appl. Surf. Sci..

[cit7] Liu X., Zhao X., Ding C., Chu P. K. (2006). Light-induced bioactive TiO_2_ surface. Appl. Phys. Lett..

[cit8] Ferraris S. (2020). *et al.*, Bioactive materials: In vitro investigation of different mechanisms of hydroxyapatite precipitation. Acta Biomater..

[cit9] Gou H., Zhao X., Liu X., Zhang T., Ding C. (2005). Study on the self-setting property and the in vitro bioactivity of β-Ca_2_SiO_4_. J. Biomed. Mater. Res., Part B.

[cit10] Taylor H. F. W. (1984). The chemistry of dicalcium silicate mineral. J. Mater. Sci..

[cit11] Jawad Ahmed M., Schollbach K., van der Laan S., Florea M., Brouwers H. J. H. (2022). A quantitative analysis of dicalcium silicate synthesized via different sol-gel methods. Mater. Des..

[cit12] Tan Y. N., Liu Y., Qing Z., Birdi G., Grover L. M. (2014). Synthesis of pure dicalcium silicate powder by the Pechini method and characterization of hydrated cement. Mater. Sci. Forum.

[cit13] Zhong H. (2011). *et al.*, Mechanical properties and bioactivity of β-Ca_2_SiO_4_ ceramics synthesized by spark plasma sintering. Ceram. Int..

[cit14] Georgescu M., Tipan J., Badanoiu A., Crisan D., Dragan I. (2000). Highly reactive dicalcium silicate synthesised by hydrothermal processing. Cem. Concr. Compos..

[cit15] El-Kheshen A. A., Khaliafa F. A., Saad E. A., Elwan R. L. (2008). Effect of Al_2_O_3_ addition on bioactivity, thermal and mechanical properties of some bioactive glasses. Ceram. Int..

[cit16] Liu W. (2019). *et al.*, Strontium-substituted dicalcium silicate bone cements with enhanced osteogenesis potential for orthopaedic applications. Materials.

[cit17] Ahmed M. J. (2023). *et al.*, V and Cr substitution in dicalcium silicate under oxidizing and reducing conditions – Synthesis, reactivity, and leaching behavior studies. J. Hazard. Mater..

[cit18] Wang X., Xie Z., Huo Z., Shi Q., Luo H., Ji S. (2025). Effect of boron doping and cooling condition on polymorph and early strength of dicalcium silicate. Constr. Build. Mater..

[cit19] Zhao M., Wang F., Liu Z., Hu S. (2024). Impact of Fe doping on the reactivity of γ-dicalcium silicate: Insights
from DFT calculations. Constr. Build. Mater..

[cit20] Chi L. (2020). *et al.*, Hydration activity, crystal structural, and electronic properties studies of Ba-doped dicalcium silicate. Nanotechnol. Rev..

[cit21] Cheng S., Shevchenko M., Hayes P. C., Jak E. (2021). Experimental phase equilibria studies in the FeO-Fe_2_O_3_-CaO-SiO_2_ system and the subsystems CaO-SiO_2_ and FeO-Fe_2_O_3_-SiO_2_ in air. Metall. Mater. Trans. B.

[cit22] Zhang D. (2024). *et al.*, The influence of boron doping on the structures and composition of dicalcium silicate: A research study. Buildings.

[cit23] Gudkov S. V., Burmistrov D. E., Smirnova V. V., Semenova A. A., Lisitsyn A. B. (2022). A mini review of antibacterial properties of Al_2_O_3_ nanoparticles. Nanomaterials.

[cit24] Sangaiya P., Jayaprakash R. (2018). A review on iron oxide nanoparticles and their biomedical applications. J. Supercond. Novel Magn..

[cit25] Mohamed A. T. (2024). Facile synthesis of Fe_2_O_3_, Fe_2_O_3_@CuO and WO_3_ nanoparticles: characterization, structure determination and evaluation of their biological activity. Sci. Rep..

[cit26] Pourmadadi M. (2022). Role of iron oxide (Fe_2_O_3_) nanocomposites in advanced biomedical applications: A state-of-the-art review. Nanomaterials.

[cit27] Pourmadadi M., Ahmadi M. J., Dinani H. S., Ajalli N., Dorkoosh F. (2022). Theranostic applications of stimulus-responsive systems based on Fe_2_O_3_. Pharm. Nanotechnol..

[cit28] Qiao Z., Shi X. (2015). Dendrimer-based molecular imaging contrast agents. Prog. Polym. Sci..

[cit29] Rahdar A., Taboada P., Aliahmad M., Hajinezhad M. R., Sadeghfar F. (2018). Iron oxide nanoparticles: Synthesis, physical characterization, and intraperitoneal biochemical studies in Rattus norvegicus. J. Mol. Struct..

[cit30] O'Connor C., Brady E., Zheng Y., Moore E., Stevens K. R. (2022). Engineering the multiscale complexity of vascular networks. Nat. Rev. Mater..

[cit31] Koons G. L., Diba M., Mikos A. G. (2020). Materials design for bone-tissue engineering. Nat. Rev. Mater..

[cit32] Ikram H., Al Rashid A., Koç M. (2022). Synthesis and characterization of hematite (α-Fe_2_O_3_) reinforced polylactic acid (PLA) nanocomposites for biomedical applications. Compos., Part C: Open Access.

[cit33] Rafi M. M., Ahmed K. S. Z., Nazeer K. P., Siva Kumar D., Thamilselvan M. (2015). Synthesis, characterization and magnetic properties of hematite (α-Fe_2_O_3_) nanoparticles on polysaccharide templates and their antibacterial activity. Appl. Nanosci..

[cit34] Yoonus J., Resmi R., Beena B. (2021). Evaluation of antibacterial and anticancer activity of green synthesized iron oxide (α-Fe_2_O_3_) nanoparticles. Mater. Today: Proc..

[cit35] Bhushan M., Muthukamalam S., Sudharani S., Viswanath A. K. (2015). Synthesis of α-Fe_2_−xAgxO_3_ nanocrystals and study of their optical, magnetic and antibacterial properties. RSC Adv..

[cit36] Bhushan M., Kumar Y., Periyasamy L., Viswanath A. K. (2018). Facile synthesis of Fe/Zn oxide nanocomposites and study of their structural, magnetic, thermal, antibacterial and cytotoxic properties. Mater. Chem. Phys..

[cit37] Bhushan M., Kumar Y., Periyasamy L., Viswanath A. K. (2018). Antibacterial applications of α-Fe_2_O_3_/Co_3_O_4_ nanocomposites and study of their structural, optical, magnetic and cytotoxic characteristics. Appl. Nanosci..

[cit38] Bhushan M., Kumar Y., Periyasamy L., Viswanath A. K. (2019). Study of synthesis, structural, optical and magnetic characterizations of iron/copper oxide nanocomposites: A promising
novel inorganic antibiotic. Mater. Sci. Eng. C.

[cit39] Bhushan M., Kumar Y., Periyasamy L., Viswanath A. K. (2019). Fabrication and a detailed study of antibacterial properties of α-Fe_2_O_3_/NiO nanocomposites along with their structural, optical, thermal, magnetic and cytotoxic features. Nanotechnology.

[cit40] Bhushan M., Mohapatra D., Kumar Y., Viswanath A. K. (2021). Fabrication of novel bioceramic α-Fe_2_O_3_/MnO nanocomposites: Study of their structural, magnetic, biocompatibility and antibacterial properties. Mater. Sci. Eng., B.

[cit41] Shareef H., Kareem Z., Alkaim A. (2018). *et*, Evaluation of antibacterial activity of Fe2O3 nanoparticles against Shigella dysenteriae. J. Pharmaceut. Sci. Res..

[cit42] Sasaki K., Hayashi Y., Toshiyuki K., Guo B. (2018). Simultaneous immobilization of borate, arsenate, and silicate from geothermal water derived from mining activity by co-precipitation with hydroxyapatite. Chemosphere.

[cit43] Hoppe A., Güldal N. S., Boccaccini A. R. (2011). A review of the biological response to ionic dissolution products from bioactive glasses and glass-ceramics. Biomaterials.

[cit44] Bai N. (2021). *et al.*, Effect of B_2_O_3_ on the structural and in vitro biological assessment of mesoporous bioactive glass nanospheres. J. Am. Ceram. Soc..

[cit45] Eviş Z. (2020). Boron doped hydroxyapatites in biomedical applications. J. Boron.

[cit46] Sayin Z., Ucan U. S., Sakmanoglu A. (2016). Antibacterial and antibiofilm effects of boron on different bacteria. Biol. Trace Elem. Res..

[cit47] Sopcak T. (2023). *et al.*, Physico-chemical, mechanical and antibacterial properties of the boron modified biphasic larnite/bredigite cements for potential use in dentistry. Ceram. Int..

[cit48] Gold K., Slay B., Knackstedt M., Gaharwar A. K. (2018). Antimicrobial activity of metal and metal-oxide based nanoparticles. Adv. Ther..

[cit49] Lee L.-H., Ha J.-S. (2014). Deposition behavior and characteristics of hydroxyapatite coatings on Al_2_O_3_, Ti, and Ti6Al4V formed by a chemical bath method. Ceram. Int..

[cit50] Xie H., Zhang L., Xu E., Yuan H., Zhao F., Gao J. (2019). SiAlON–Al_2_O_3_ ceramics as potential biomaterials. Ceram. Int..

[cit51] Tripathi H., Hira S. K., Kumar A. S., Gupta U., Manna P. P., Singh S. P. (2015). Structural characterization and in vitro bioactivity assessment of SiO_2_–CaO–P_2_O_5_–K_2_O–Al_2_O_3_ glass as bioactive ceramic material. Ceram. Int..

[cit52] Ghelani D., Faisal S. (2018). Synthesis and characterization of aluminium oxide nanoparticles. Int. J. Pharm. Pharm. Sci..

[cit53] Kumar A., Kandpal M. (2023). Synthesis, characterization, and antibacterial activity of biocompatible aluminium complexes with N-(2-hydroxy-5-methyl benzyl)phenylalanine. J. Chem..

[cit54] Wu Y., Hu C. C., Liu B., Huang Y. H., Song K. X. (2020). Crystal structure, vibrational spectroscopy, and microwave dielectric properties of CaAl_4_O_7_ ceramics with low permittivity. J. Mater. Sci.: Mater. Electron..

[cit55] Puchalska M., Gerasymchuk Y., Zych E. (2010). Optical properties of Eu^3+^-doped CaAl_4_O_7_ synthesized by the Pechini method. Opt. Mater..

[cit56] Panda R., Behera M., Kumar R. A., Joshi D., Padhi R. K. (2023). Luminescence studies of high color purity red-emitting CaAl_4_O_7_:Eu^3+^ phosphor prepared by microwave-assisted synthesis technique. J. Alloys Compd..

[cit57] Fusayama P., Katayori T., Nomoto S. (1963). About a synthetic saliva for in vitro studies. Bull. Tokyo Med. Dent. Univ..

[cit58] Deliormanlı A. M. (2017). Investigation of in vitro mineralization of silicate-based 45S5 and 13-93 bioactive glasses in artificial saliva for dental applications. Ceram. Int..

[cit59] Safwat E. M. (2023). *et al.*, Preparation and characterization of dental pit and fissure sealant based on calcium sodium silicate bioactive glasses. Silicon.

[cit60] Holopainen J., Ritala M. (2016). Rapid production of bioactive hydroxyapatite fibers via electroblowing. J. Eur. Ceram. Soc..

[cit61] Kokubo T., Takadama H. (2006). How useful is SBF in predicting in vivo bone bioactivity?. Biomaterials.

[cit62] Juhasz J. A., Best S. M., Auffret A. D., Bonfield W. (2008). Biological control of apatite growth in simulated body fluid and human blood serum. J. Mater. Sci.: Mater. Med..

[cit63] Oyane A., Kim H.-M., Furuya T., Kokubo T., Miyazaki T., Nakamura T. (2003). Preparation and assessment of revised simulated body fluids. J. Biomed. Mater. Res., Part A.

[cit64] Rad M. M., Babaki A., Aghdam M. M., Saber-Samandari S., Sadighi M. (2026). A study on mechanical performance and bioactivity of PMMA-HA-graphene bone cements. Mater. Today Commun..

[cit65] López R., Gómez R. (2012). Band-gap energy estimation from diffuse reflectance measurements on sol–gel and commercial TiO_2_: A comparative study. J. Sol-Gel Sci. Technol..

[cit66] Sun J., Wang H., Zhang Y., Zheng Y., Xu Z., Liu R. (2014). Structure and luminescent properties of electrodeposited Eu^3+^-doped CaF_2_ thin films. Thin Solid Films.

[cit67] Punj S., Singh K. (2019). Blue-green light emitting inherent luminescent glasses synthesized from agro-food wastes. J. Mater. Sci.: Mater. Electron..

[cit68] Singh V., Kaur S., Jayasimhadri M. (2020). Luminescence properties of orange emitting CaAl_4_O_7_:Sm^3+^ phosphor for solid state lighting applications. Solid State Sci..

[cit69] Dhankhar S., Bhalerao G., Ganesamoorthy S., Baskar K., Singh S. (2017). Growth and comparison of single crystals and polycrystalline brownmillerite Ca_2_Fe_2_O_5_. J. Cryst. Growth.

[cit70] Hamdaoui O., Naffrechoux E. (2007). Modeling of adsorption isotherms of phenol and chlorophenols onto granular activated carbon: Part I. Two-parameter models and equations allowing determination of thermodynamic parameters. J. Hazard. Mater..

[cit71] Escalante-García J. I., Sharp J. H., Mendoza-Suarez E. (2009). An FTIR, SEM and EDS investigation of solidification/stabilization of chromium using Portland cement Type V and Type IP. J. Hazard. Mater..

[cit72] Yin H. (2018). *et al.*, Fabrication and characterization of strontium-doped borate-based bioactive glass scaffolds for bone tissue engineering. J. Alloys Compd..

[cit73] Mhashakhetri S. T., Yerpude A. N., Nandanwar C. M., Nande A. V., Dhoble S. J. (2021). Investigation of structural, microstructural and optical properties of Ca_3_B_2_O_6_:Sm^3+^ phosphor. J. Mater. Sci.: Mater. Electron..

[cit74] Charak I. (2021). *et al.*, Structural and spectral studies of highly pure red-emitting Ca_3_B_2_O_6_:Eu^3+^ phosphors for white light emitting diodes. J. Alloys Compd..

[cit75] Benallal S., Boumaza S., Brahimi R., Trari M. (2022). Elaboration and characterization of the brownmillerite Ca_2_Fe_2_O_5_ synthesized by citrate sol-gel method. Application for photocatalytic H_2_ production under visible light over Ca_2_Fe_2_O_5_/ZnO hetero-system. Int. J. Hydrogen Energy.

[cit76] Wang L. G., Zeng P. Y., Zhu C. M., Yu G. B., Cui H., Wang R. (2023). Insight into the low-temperature electrical polarization behavior of multiferroic Bi_2_Fe_4_O_9_. Ceram. Int..

[cit77] Timón V., Torrens-Martin D., Fernández-Carrasco L. J., Martínez-Ramírez S. (2023). Infrared and Raman vibrational modelling of β-C_2_S and C_3_S compounds. Cem. Concr. Res..

[cit78] Juhim F. (2023). *et al.*, Study of gamma radiation shielding on tellurite glass containing TiO_2_ and Al_2_O_3_ nanoparticles. Heliyon.

[cit79] Seshadri P. K. C., Parameshwarappa S. B., Shetty V. R., Sivasankara Reddy N. R. (2024). Structural and optical properties of alumino lead borate glasses containing copper oxide. Trans. Indian Ceram. Soc..

[cit80] Singh V., Boddula R., Nikhare G. N., Joo J. B. (2022). Orange-red luminescence features of Eu^3+^ doped CaAl_4_O_7_ phosphors. Optik.

[cit81] Qi S., Huang Y., Lin Q., Cheng H., Seo H. J. (2015). A bioactive Ca_2_SiB_2_O_7_ ceramics and in vitro hydroxyapatite mineralization ability in SBF. Ceram. Int..

[cit82] Beyli P. T., Doğan M., Gündüz Z., Alkan M., Turhan Y. (2018). Synthesis, characterization and their antimicrobial activities of boron oxide/poly(acrylic acid) nanocomposites: Thermal and antimicrobial properties. Adv. Mater. Sci..

[cit83] Tetteh G., Khan A. S., Delaine-Smith R. M., Reilly G. C., Rehman I. U. (2014). Electrospun polyurethane/hydroxyapatite bioactive scaffolds for bone tissue engineering: The role of solvent and hydroxyapatite particles. J. Mech. Behav. Biomed. Mater..

[cit84] De Santis R. (2015). *et al.*, Towards the design of 3D fiber-deposited poly(ε-caprolactone)/iron-doped hydroxyapatite nanocomposite magnetic scaffolds for bone regeneration. J. Biomed. Nanotechnol..

[cit85] Wu Y., Hench L. L., Du J., Choy K., Guo J. (2004). Preparation of hydroxyapatite fibers by electrospinning technique. J. Am. Ceram. Soc..

[cit86] Paşcu E. I., Stokes J., McGuinness G. B. (2013). Electrospun composites of PHBV, silk fibroin and nano-hydroxyapatite for bone tissue engineering. Mater. Sci. Eng. C.

[cit87] Ranjan Dev P., Parambil Anand C., Samuvel Michael D., Wilson P. (2022). Hydroxyapatite coatings: A critical review on electrodeposition parametric variations influencing crystal facet orientation towards enhanced electrochemical sensing. Mater. Adv..

[cit88] Qidwai M., Sheraz M. A., Ahmed S., Alkhuraif A. A., ur Rehman I. (2014). Preparation and characterization of bioactive composites and fibers for dental applications. Dent. Mater..

[cit89] Sayin Z., Ucan U. S., Sakmanoglu A. (2016). Antibacterial and antibiofilm effects of boron on different bacteria. Biol. Trace Elem. Res..

[cit90] Sarac N., Ugur A., Boran R., Elgin E. S. (2015). The use of boron compounds for stabilization of lipase from Pseudomonas aeruginosa ES3 for the detergent industry. J. Surfactants Deterg..

[cit91] Zan R., Hubbezoglu I., Ozdemır A., Tunc T., Sumer Z., Alıcı O. (2014). Antibacterial effect of different concentration of boric acid against Enterococcus faecalis biofilms in root canal. Marmara Dent. J..

[cit92] Bhuvaneshwari M., Bairoliya S., Parashar A., Chandrasekaran N., Mukherjee A. (2016). Differential toxicity of Al_2_O_3_ particles on Gram-positive and Gram-negative sediment bacterial isolates from freshwater. Environ. Sci. Pollut. Res..

[cit93] Kamel R. M., Yaaqoob L. A. (2022). Evaluation of the biological effect of synthesized iron oxide nanoparticles on Enterococcus faecalis. Iraqi J. Agric. Sci..

[cit94] Al-Mallah S., Al-Naimi A. M. (2021). Minimum inhibitory concentration of iron oxide nanoparticles with hydrogen peroxide against endodontic Enterococcus faecalis. Al-Rafidain Dent. J..

[cit95] Shahbazi E., Morshedzadeh F., Zaeifi D. (2019). Bacteriostatic potency of Fe_2_O_3_ against Enterococcus faecalis in synergy with antibiotics by DDST method. Avicenna J. Med. Biotechnol..

[cit96] Armijo L. M. (2020). *et al.*, Antibacterial activity of iron oxide, iron nitride, and tobramycin conjugated nanoparticles against Pseudomonas aeruginosa biofilms. J. Nanobiotechnol..

[cit97] Gholami A., Mohammadi F., Ghasemi Y., Omidifar N., Ebrahiminezhad A. (2020). Antibacterial activity of SPIONs versus ferrous and ferric ions under aerobic and anaerobic conditions: A preliminary mechanism study. IET Nanobiotechnol..

[cit98] Zhang J. (2026). *et al.*, Zeolite-confined ionic liquid enables on-demand active bromine generation for thermally robust and reduced-dosage antimicrobial materials. Ind. Chem. Mater.

[cit99] Saliani M., Jalal R., Goharshadi E. K. (2015). Effects of pH and temperature on antibacterial activity of zinc oxide nanofluid against Escherichia coli O157:H7 and Staphylococcus aureus. Jundishapur J. Microbiol..

[cit100] Yuan Y., Ding S., Li C., Wang X., Xu J., Xia K. (2018). Effect of local alkaline microenvironment on the behaviors of bacteria and osteogenic cells. ACS Appl. Mater. Interfaces.

[cit101] Rahim M. I., Eifler R., Rais B., Mueller P. P. (2015). Alkalization is responsible for antibacterial effects of corroding magnesium. J. Biomed. Mater. Res., Part A.

[cit102] Okpara E. C., Wojuola O. B., Fayemi O. E., Oyewo O. A., Onwudiwe D. C. (2022). Sol–gel synthesis and electrochemical sensing properties of brownmillerite calcium ferrite–Ca_2_Fe_2_O_5_ nanoparticles. J. Inorg. Organomet. Polym. Mater..

[cit103] Nagabhushana H. (2010). Thermoluminescence and defect study of MgSiO_3_ ceramics. Philos. Mag.

[cit104] Smitha J. K., Bhaskar S. P., Jose A., Geetha T. (2024). Synthesis of nano calcium borate using sugarcane bagasse extract as a capping agent and its potential to promote germination in cowpea seeds. Results Chem..

[cit105] Meier S. (2020). *et al.*, Synthesis of calcium borate nanoparticles and its use as a potential foliar fertilizer in lettuce (Lactuca sativa) and zucchini (Cucurbita pepo). Plant Physiol. Biochem..

[cit106] Erfani M., Saion E., Soltani N., Hashim M., Abdullah W. S. B. W., Navasery M. (2012). Facile synthesis of calcium borate nanoparticles and the annealing effect on their structure and size. Int. J. Mol. Sci..

[cit107] Karmaoui M., Willinger M.-G., Mafra L., Herntrich T., Pinna N. (2009). A general nonaqueous route to crystalline alkaline earth aluminate nanostructures. Nanoscale.

[cit108] Mitchell B. S. (2001). Nanocrystallinity in heat-treated calcium aluminate fibers. Mater. Lett..

[cit109] Prasittisopin L. (2025). Nanotechnology for calcium aluminate cement: Thematic analysis. Rev. Adv. Mater. Sci..

[cit110] Bohner M., Lemaitre J. (2009). Can bioactivity be tested in vitro with SBF solution?. Biomaterials.

[cit111] Shuai C. (2017). *et al.*, Calcium silicate improved bioactivity and mechanical properties of poly(3-hydroxybutyrate-co-3-hydroxyvalerate) scaffolds. Polymers.

[cit112] Hench L. L. (2006). The story of Bioglass®. J. Mater. Sci.: Mater. Med..

[cit113] Saravana Karthikeyan S. K. S. (2018). *et al.*, Understanding of the elastic constants, energetics, and bonding in dicalcium silicate using first-principles calculations. J. Phys. Chem. C.

[cit114] Dadashov R., Voves J. (2024). The atomistic model of electronic properties of Al_2_O_3_ and ZnO for the calculations of Al-doped ZnO. J. Phys.: Conf. Ser..

[cit115] Wang Z. P., Ching W. Y. (1996). Electronic structures and optical properties of low- and high-pressure phases of crystalline BeO. Phys. Rev. B.

[cit116] Mallick P. (2014). Influence of different materials on the microstructure and optical band gap of α-Fe_2_O_3_ nanoparticles. Mater. Sci.-Pol..

